# T cell memory revisited using single telomere length analysis

**DOI:** 10.3389/fimmu.2023.1100535

**Published:** 2023-09-14

**Authors:** Laureline Roger, Kelly L. Miners, Louise Leonard, Julia W. Grimstead, David A. Price, Duncan M. Baird, Kristin Ladell

**Affiliations:** ^1^ Division of Infection and Immunity, Cardiff University School of Medicine, University Hospital of Wales, Cardiff, United Kingdom; ^2^ Division of Cancer and Genetics, Cardiff University School of Medicine, University Hospital of Wales, Cardiff, United Kingdom; ^3^ Systems Immunity Research Institute, Cardiff University School of Medicine, University Hospital of Wales, Cardiff, United Kingdom

**Keywords:** replicative history, T cell differentiation, T cell memory, T cell senescence, telomere length (TL)

## Abstract

The fundamental basis of T cell memory remains elusive. It is established that antigen stimulation drives clonal proliferation and differentiation, but the relationship between cellular phenotype, replicative history, and longevity, which is likely essential for durable memory, has proven difficult to elucidate. To address these issues, we used conventional markers of differentiation to identify and isolate various subsets of CD8^+^ memory T cells and measured telomere lengths in these phenotypically defined populations using the most sensitive technique developed to date, namely single telomere length analysis (STELA). Naive cells were excluded on the basis of dual expression of CCR7 and CD45RA. Memory subsets were sorted as CD27^+^CD45RA^+^, CD27^int^CD45RA^+^, CD27^−^CD45RA^+^, CD27^+^CD45RA^int^, CD27^−^CD45RA^int^, CD27^+^CD45RA^−^, and CD27^−^CD45RA^−^ at >98% purity. The shortest median telomere lengths were detected among subsets that lacked expression of CD45RA, and the longest median telomere lengths were detected among subsets that expressed CD45RA. Longer median telomere lengths were also a feature of subsets that expressed CD27 in compartments defined by the absence or presence of CD45RA. Collectively, these data suggested a disconnect between replicative history and CD8^+^ memory T cell differentiation, which is classically thought to be a linear process that culminates with revertant expression of CD45RA.

## Introduction

The ability to remember previous antigen encounters is a hallmark of adaptive immunity (1). Our basic understanding of immunological memory nonetheless remains incomplete, especially for helper T cells, which classically express CD4, and effector T cells, which classically express CD8. In each of these lineages, naive cells undergo clonal proliferation and differentiation in response to antigen stimulation, generating a spectrum of daughter cells that populate a functionally and phenotypically heterogeneous memory landscape (2–4). However, the relationship between cellular phenotype, replicative history, and longevity, which is likely essential for durable memory, has proven difficult to elucidate.

Adoptive transfer studies have shown that stem cell-like memory T (T_SCM_) and central memory T (T_CM_) cells persist *in vivo* and establish durable protection more readily than effector memory T (T_EM_) cells (5–7). These observations have been linked with the ability of T_SCM_ and T_CM_ cells to self-renew and proliferate to a greater extent than T_EM_ cells in response to antigen stimulation (6, 8, 9). In turn, such proliferative reserve is thought to reflect fewer antecedent cell divisions, consistent with a less differentiated phenotype characterized by the expression of various costimulatory molecules, such as CD27 and CD28, and chemokine/cytokine receptors, such as CCR7 and CD127 (10, 11).

Replicative history as a metric of cellular age can be assessed by measuring telomere lengths. Telomeres are nucleoprotein complexes that cap the ends of linear chromosomes and undergo division-linked erosion due to incomplete synthesis of the lagging strand during semiconservative DNA replication (12), coupled with the functional requirement for a terminal 3′ single-stranded G-rich overhang generated by nucleolytic activity. Consequently, telomeres in human cells shorten progressively with ongoing cell division in the absence of telomerase, which compensates to some extent for deficiencies in the DNA replication machinery. This process ultimately limits the replicative lifespan of any cell when telomeres shorten to a critical length, triggering a state of retinoblastoma tumor suppressor protein (RB)-dependent and tumor protein p53 (TP53)-dependent cell cycle arrest known as replicative senescence (13).

Telomere lengths have been assessed previously among immune cell subsets using hybridization-based techniques, such as terminal restriction fragment (TRF) analysis and fluorescence *in situ* hybridization via flow cytometry (flowFISH), and polymerase chain reaction (PCR)-based techniques, such as the telomere shortest length assay (TeSLA) (14–24). However, these approaches have various limitations that render them potentially unsuitable for the detection of subtle differences in telomere length distributions across related cell populations, which we considered a likely scenario in the case of lineage-defined memory T cell immunity. Accordingly, we used a different approach in this study to characterize the telomere length profiles of CD8^+^ memory T cell subsets identified and isolated via polychromatic flow cytometry, namely single telomere length analysis (STELA). Our data revealed highly significant differences in terms of median telomere length that segregated with expression patterns of CD27 and CD45RA.

## Methods

### Donors

Healthy adult volunteers aged 28–48 years (n = 5) were recruited for this study. Peripheral blood mononuclear cells (PBMCs) were isolated from 50 mL of venous blood via standard density gradient centrifugation using Histopaque 1077 (Sigma-Aldrich). Approval was granted by the Cardiff University School of Medicine Research Ethics Committee (12/09). Informed consent was obtained from all donors in accordance with the principles of the Declaration of Helsinki.

### Flow cytometry

T cell subsets of interest were flow-sorted from freshly isolated PBMCs at >98% purity using a modified FACSAria II (BD Biosciences). Cells were stained with the following reagents: (i) anti-CD3–APC-H7 (clone SK7), anti-CD14–V500 (clone M5E2), anti-CD19–V500 (clone HIB19), anti-CD28–APC (clone CD28.2), anti-CD45RA–PE (clone HI100), anti-CD57–FITC (clone NK-1), and anti-CCR7–PE-Cy7 (clone 3D12) from BD Biosciences; (ii) anti-CD4–PE-Cy5.5 (clone S3.5), anti-CD27–QD605 (clone CLB-27/1), and LIVE/DEAD Fixable Aqua from Thermo Fisher Scientific; (iii) anti-CD8–BV711 (clone RPA-T8), anti-CD95–PE-Cy5 (clone DX2), and anti-CD127–BV421 (clone A019D5) from BioLegend; and (iv) anti-CD45RO–ECD (clone UCHL1) from Beckman Coulter. Viable memory T cells were identified in the CD8^+^ lineage after gating out naive events on the basis of dual expression of CCR7 and CD45RA. Data were analyzed using FACSDiva software version 8.0 (BD Biosciences) and FlowJo software version 9.9.6 (FlowJo LLC).

### Single telomere length analysis

DNA was extracted from 3,000 flow-sorted T cells per subset using a QIAmp DNA Micro Kit (Qiagen). STELA was carried out at the XpYp and 17p telomeres as described previously (25). For each sample, 1 µM of the Telorette-2 linker was added to purified genomic DNA in a final volume of 40 µL. Multiple PCRs were performed for each test DNA in volumes of 10 µL incorporating 1 µL of the DNA/Telorette-2 mix and 0.5 µM of the telomere-adjacent and Teltail primers in 75 mM Tris-HCl pH 8.8, 20 mM (NH_4_)_2_SO_4_, 0.01% Tween-20, and 1.5 mM MgCl_2_, with 0.5 U of a 10:1 mixture of Taq (ABGene) and Pwo polymerase (Roche Molecular Biochemicals). The reactions were processed in a Tetrad2 Thermal Cycler (Bio-Rad). DNA fragments were resolved using 0.5% Tris-acetate-EDTA agarose gel electrophoresis and identified via Southern hybridization using a random-primed α-^33^P-labeled (PerkinElmer) TTAGGG repeat probe, together with probes specific for the 1 kb (Stratagene) and 2.5 kb molecular weight markers (Bio-Rad). Hybridized fragments were detected using a Typhoon FLA 9500 Phosphorimager (GE Healthcare). The molecular weights of the DNA fragments were calculated using a Phoretix 1D Quantifier (Nonlinear Dynamics).

### Statistics

Telomere lengths were compared across memory T cell populations using the Kruskal-Wallis test with Dunn’s *post-hoc* test for multiple comparisons in Prism software version 7 (GraphPad). Pooled data were analyzed similarly in R. Significance was assigned at p < 0.05.

## Results

**Figure 1:**
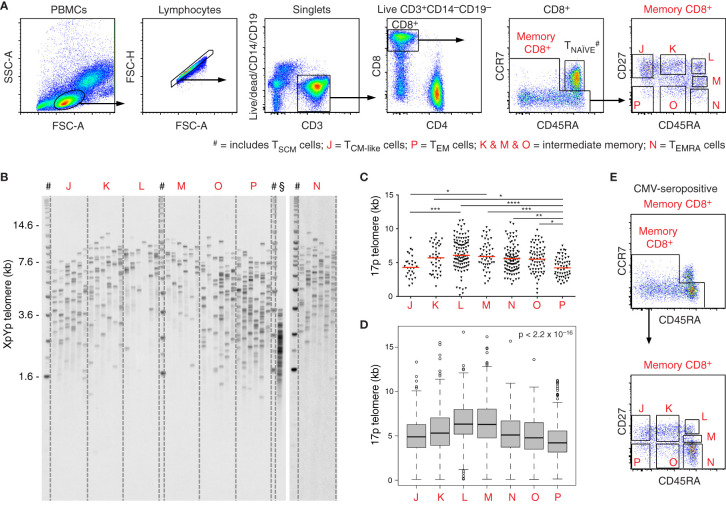
Telomere length distributions among CD8^+^ memory T cell subsets revealed using STELA. **(A)** Flow cytometric gating strategy. Populations labeled J–P were flow-sorted at >98% purity for telomere length assessment via STELA. Data from donor 1. **(B)** Southern blot showing XpYp telomere length data for the subsets in **(A)**. ^#^DNA ladder; ^§^fibroblasts (control). **(C)** Scatter plot depicting 17p telomere length distributions for the subsets in **(A)**. Horizontal red lines indicate median values. *p < 0.05, **p < 0.01, ***p < 0.001, ****p < 0.0001 (Kruskal-Wallis test with Dunn’s *post-hoc* test for multiple comparisons). **(D)** Boxplot depicting pooled telomere length distributions (n = 5 donors). Significance was assessed using the Kruskal-Wallis test. Individual comparisons and corrected values are shown in **Table 1**. **(E)** Flow cytometry plots showing the distribution of CD8^+^ memory T cells according to expression levels of CD27 and CD45RA in the presence of CMV. Data from donor 4.

To explore the relationship between differentiation and replicative history in the peripheral CD8^+^ T cell lineage, we processed freshly drawn venous blood samples from healthy volunteers (n = 5) and isolated phenotypically defined memory subsets (n = 7) characterized using polychromatic flow cytometry (**Figure 1A**). Naive cells were excluded on the basis of dual expression of CCR7 and CD45RA. Memory subsets were sorted as CD27^+^CD45RA^+^, CD27^int^CD45RA^+^, CD27^−^CD45RA^+^, CD27^+^CD45RA^int^, CD27^−^CD45RA^int^, CD27^+^CD45RA^−^, and CD27^−^CD45RA^−^ at >98% purity. Telomere length distributions were then characterized for each subset using STELA (**Figure 1B**). Importantly, similar length distributions were observed for the XpYp and 17p telomeres across all CD8^+^ memory T cell subsets, highlighting the reproducibility of data obtained using this approach (**Supplementary Figures 1A, B**).

**Table 1:** Median telomere lengths for naive and memory cells in the CD8^+^ T cell lineage.

Donor	J	K	L	M	N	O	P	CCR7^+^CD45RA^+^
**1**	4.29	5.71	6.07	5.89	5.37	5.51	4.22	6.57
**2**	5.12	5.56	6.43	**7.19**	6.58	4.52	4.57	6.81
**3**	5.62	5.65	6.99	**7.13**	5.99	5.19	5.16	7.00
**4**	3.88	4.81	**5.67**	4.43	3.70	3.59	3.40	5.35
**5**	5.40	5.05	**7.92**	7.39	5.66	5.65	5.09	7.59

Bold font denotes values above those observed for naive cells (CCR7^+^CD45RA^+^).

In line with the known heterogeneity of memory T cells in the vascular circulation, we observed considerable overlap across the telomere length distributions acquired from subsets with distinct expression levels of CD27 and CD45RA. Intersubset differences were nonetheless apparent in terms of spread and median telomere length (**Figures 1C, D**). Counterintuitively, the shortest median telomere lengths were displayed by memory subsets lacking expression of CD45RA, especially in the absence of CD27 (**Figures 1B–D**).

**Figure 2:**
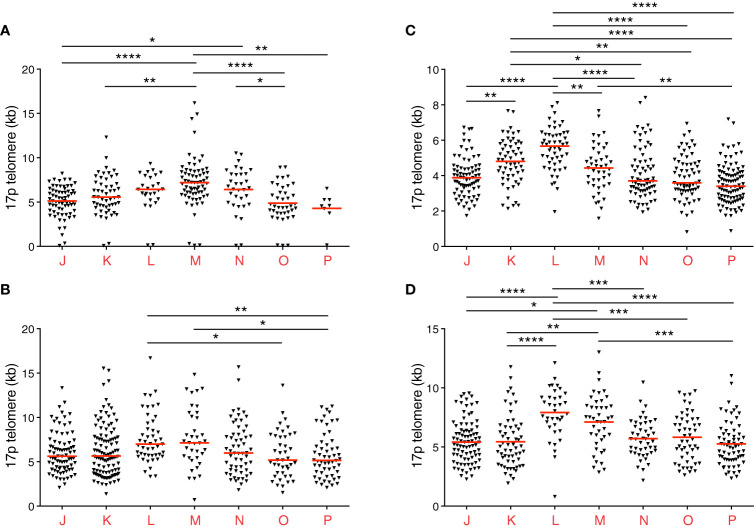
Individual telomere length distribution patterns among CD8^+^ memory T cell subsets revealed using STELA. **(A–D)** Scatter plots depicting 17p telomere length distributions from donors 2 **(A)**, 3 **(B)**, 4 **(C)**, and 5 **(D)**. Horizontal red lines indicate median values. *p < 0.05, **p < 0.01, ***p < 0.001, ****p < 0.0001 (Kruskal-Wallis test with Dunn’s *post-hoc* test for multiple comparisons). Donors 4 and 5 were known to be seropositive for CMV.

Of note, donor 2 was known to be seronegative for cytomegalovirus (CMV), and donors 4 and 5 were known to be seropositive for CMV. In donors 4 and 5, high frequencies of CD8^+^ memory T cells expressed CD45RA (**Figure 1E**), as expected in the presence of CMV. Telomere lengths in this compartment also paralleled the expression of CD27 more closely in donors 4 and 5 compared with donors 1, 2, and 3 (**Figures 1C**, **2A–D**).

Closer inspection of the data further revealed interindividual variations in telomere length (**Figures 1C**, **2A–D** and **Table 1**). For example, the shortest median telomere length in donor 3 was 5.16 kb for the CD27^−^CD45RA^−^ subset (population P) (**Figure 2B**), and the shortest median telomere length in donor 4 was 3.4 kb for the CD27^−^CD45RA^−^ subset (population P) (**Figure 2C**). In contrast, the longest median telomere length in donor 3 was 7.13 kb for the CD27^int^CD45RA^+^ subset (population M) (**Figure 2B**), whereas the longest median telomere length in donor 4 was 5.67 kb for the CD27^+^CD45RA^+^ subset (population L) (**Figure 2C**).

**Table 2:** Pooled telomere length data analyzed using Dunn’s *post-hoc* test for multiple comparisons.

T cell subsets	Mean rank difference	Adjusted p value
**J vs. K**	−127.8	0.0922
**J vs. L**	−392	<0.0001
**J vs. M**	−342.5	<0.0001
**J vs. N**	−67.73	>0.9999
**J vs. O**	−4.448	>0.9999
**J vs. P**	169	0.0057
**K vs. L**	−264.3	<0.0001
**K vs. M**	−214.7	0.0001
**K vs. N**	60.05	>0.9999
**K vs. O**	123.3	0.2009
**K vs. P**	296.8	<0.0001
**L vs. M**	49.55	>0.9999
**L vs. N**	324.3	<0.0001
**L vs. O**	387.6	<0.0001
**L vs. P**	561.1	<0.0001
**M vs. N**	274.8	<0.0001
**M vs. O**	338	<0.0001
**M vs. P**	511.5	<0.0001
**N vs. O**	63.28	>0.9999
**N vs. P**	236.8	<0.0001
**O vs. P**	173.5	0.0085

Alpha = 0.05.

Analysis of the pooled data confirmed that the shortest telomeres were present among CD8^+^ memory T cells lacking expression of CD27 and CD45RA (**Table 2**). It was also notable that the CD27^+^CD45RA^+^ and CD27^int^CD45RA^+^ subsets (populations L and M, respectively) in donors 2, 3, 4, and 5 harbored telomeres with median lengths approximating or even exceeding those observed among the corresponding naive subsets, defined on the basis of dual expression of CCR7 and CD45RA (**Table 1**). This observation suggested a close relationship in terms of biological age and replicative history between naive cells and memory subsets expressing CD27 and CD45RA.

It nonetheless remained conceivable that some naive cells had stained poorly for CCR7 and were consequently included in the sort gates for populations L and M, defined by the expression of CD27 and CD45RA. In line with this possibility, a detailed phenotypic analysis revealed that small fractions of cells in these populations lacked expression of the memory marker CD95 (**Supplementary Figures 2A, B**). To determine the impact of these potential contaminants on the telomere length profiles of populations L and M, we sorted additional memory subsets from the parent gates as CD57^−^CD95^+^, CD57^+^CD95^+^, and, where present, CD57^−^CD95^−^ at >98% purity. Telomere length distributions were then characterized for each subset as above using STELA (**Supplementary Figures 3A–D**). No significant differences in median telomere length were detected between the parental populations (L and M) and subsets defined according to the expression of CD95 (**Supplementary Table 1**).

Collectively, these data revealed a clear association between differentiation phenotype and telomere length across the spectrum of classically defined CD8^+^ memory T cells in the vascular circulation, which unlike current linear models, suggested that replicative history was not directly linked with revertant expression of CD45RA.

## Discussion

In this study, we used polychromatic flow cytometry and STELA to profile telomere length distributions among CD8^+^ memory T cell subsets defined according to standard phenotypic markers of differentiation, namely CD27 and CD45RA. We detected the shortest median telomere lengths among subsets that lacked expression of CD45RA and the longest median telomere lengths among subsets that expressed CD45RA. Longer median telomere lengths were also a feature of subsets that expressed CD27 in compartments defined by the absence or presence of CD45RA. These observations held after further stratification based on the expression of CD95. Collectively, our data validated a new approach to the study of immune senescence, which in turn could help illuminate the cellular processes that underlie the induction and maintenance of T cell memory.

STELA has several advantages over other techniques used to measure telomere length (14–24). First, conventional approaches measure telomere lengths across all chromosomes simultaneously, which is problematic in light of the fact that each telomere varies independently (26). Second, the hybridization-based techniques TRF and flowFISH have a lower length limit of detection, which precludes the capture of very short telomeres that characterize the extinction point of replicative capacity. TeSLA circumvents this problem by focusing on short telomeres but lacks sensitivity in terms of representing the entire length distribution (16). Third, a heating step is required for hybridization in the context of flowFISH, which alters the expression of phenotypic markers on the cell surface (18). In contrast, real-time PCR assays enable large-scale analyses without disruption to the cell surface phenotype but suffer from high measurement errors and a lack of linearity with other methods in the short telomere length range (27, 28). STELA overcomes these limitations by revealing the entire distribution of telomere lengths as well as mean/median information, thereby reducing data heterogeneity. As a single-molecule technique, STELA is also compatible with analyses of rare cell populations, including those involved in adaptive immunity (29–31).

Classical linear models propose that terminally differentiated or “end-stage” T_EM_ cells express CD45RA (32, 33). In line with this notion, previous studies using less sensitive techniques have detected shorter telomeres among these so-called T_EMRA_ cells versus T_EM_ cells that do not express CD45RA (19, 20), albeit not universally (19, 34, 35). However, other studies have shed a more positive light on CD8^+^ memory T cells that express CD45RA. For example, vaccinia virus vaccination was found to elicit long-lived memory cells that expressed intermediate levels of CD27 and lacked CD45RO (36), and yellow fever vaccination was found to elicit long-lived memory cells that expressed CCR7 and CD45RA (37). Similarly, dengue virus was found to elicit long-lived populations of CD57^+^CD127^−^ and CD57^−^CD127^+^ memory cells, both of which expressed CD45RA (38). Equivalent phenotypes have also been reported after infection with SARS-CoV-2 (39).

Another key finding of our study was that longer telomeres associated with the expression of CD27 among CD8^+^ memory T cell subsets that either expressed or did not express CD45RA. This observation is consistent with the atypical stem-like phenotypes reported after vaccination with vaccinia virus or yellow fever virus and natural infection with dengue virus or SARS-CoV-2 (36–39). Stem-like properties have also been attributed to tissue-recirculating T_EM_ cells that express CD27 (40). In line with this notion, our data suggested that replicative capacity could be assessed indirectly using the surrogate marker CD27, at least among subsets initially stratified according to the expression of CD45RA.

It should be noted that our study was limited in terms of donor numbers and further limited potentially by the fact that we did not assess telomerase activity. However, the collective data clearly showed that progressive telomere shortening was not inevitably linked with revertant expression of CD45RA, thereby challenging simple linear models of CD8^+^ memory T cell differentiation. Although further work is required to reconcile these counterintuitive observations with current paradigms in the quest for a more comprehensive understanding of immunological memory, it would seem prudent on the basis of the findings reported here to reconsider the notion that replicative senescence can be aligned with revertant expression of CD45RA.

## Data availability statement

The raw data supporting the conclusions of this article will be made available by the authors, without undue reservation.

## Ethics statement

This study was reviewed and approved by the Cardiff University School of Medicine Research Ethics Committee. All participants provided written informed consent in accordance with the principles of the Declaration of Helsinki.

## Author contributions

DP, DB, and KL designed experiments; LR, KM, JG, and KL performed experiments; DB and KL supervised experiments; LR, KM, LL, DB, and KL analyzed data; DP, DB, and KL wrote the manuscript. All authors contributed to the article and approved the submitted version.
