# Supplementary material for: T cell memory revisited using single telomere length analysis
Source: Front Immunol. 2023 Sep 14;14:1100535. doi: 10.3389/fimmu.2023.1100535 (PMC10536158; doi:10.3389/fimmu.2023.1100535)
Supplement: Supplementary file 1 [file DataSheet_1.pdf]

Supplementary Material

T cell memory revisited using single telomere length analysis

Laureline Roger[1], Kelly L. Miners[1], Louise Leonard[1], Julia W. Grimstead[2], David A. Price[1,3], Duncan M. Baird[2], Kristin Ladell[1*]

[1]Division of Infection and Immunity, Cardiff University School of Medicine, University Hospital of Wales, Cardiff CF14 4XN, UK

[2]Division of Cancer and Genetics, Cardiff University School of Medicine, University Hospital of Wales, Cardiff CF14 4XN, UK

[3]Systems Immunity Research Institute, Cardiff University School of Medicine, University Hospital of Wales, Cardiff CF14 4XN, UK

*Correspondence should be address to Kristin Ladell (ladellk@cardiff.ac.uk).

Keywords: replicative history, T cell differentiation, T cell memory, T cell senescence, telomere length (TL).

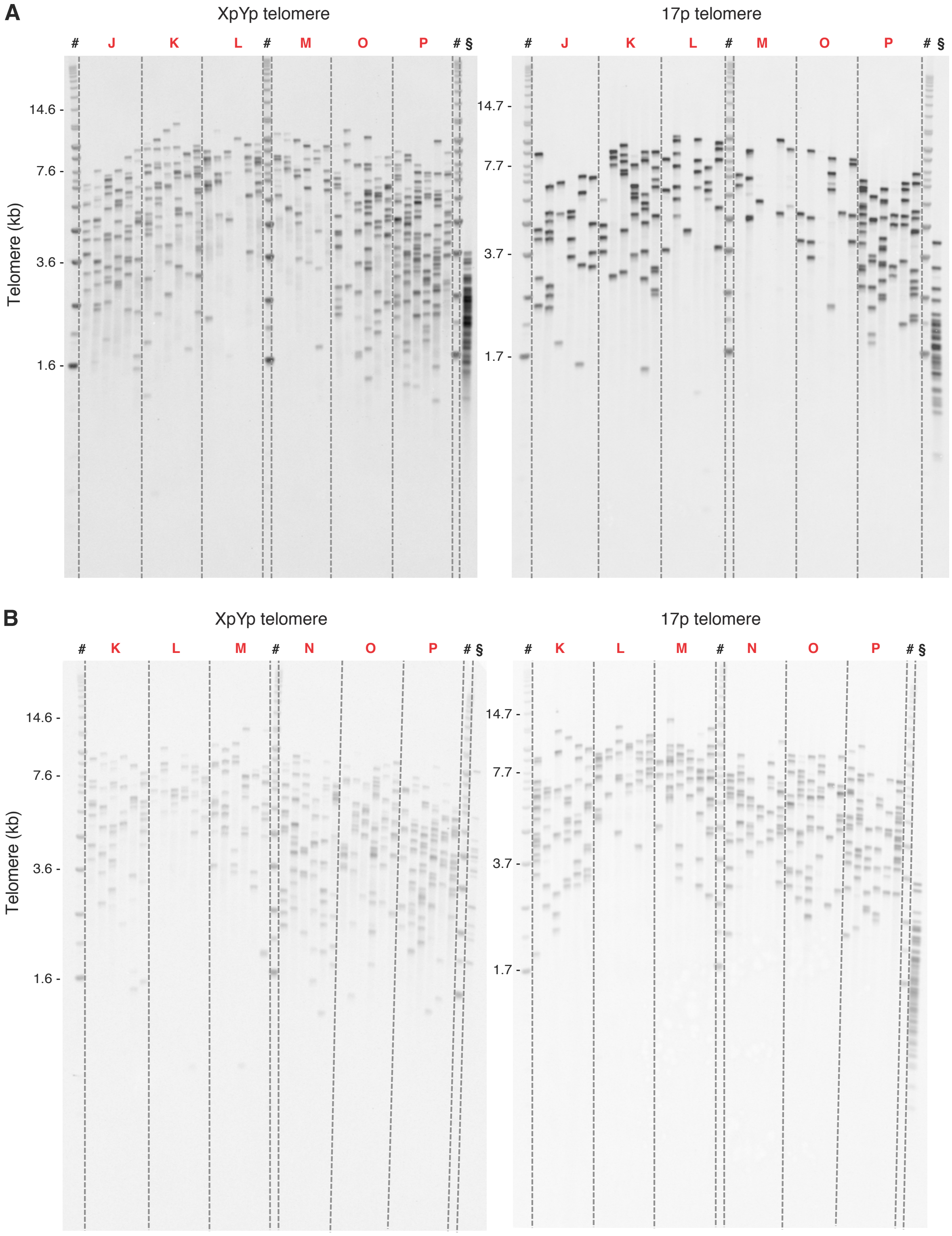

Supplementary Figure 1. Comparison of XpYp and 17p STELA. (**A**, **B**) Southern blots showing XpYp and 17p telomere length data for selected CD8^{+} memory T cell subsets from donor 1 (**A**) and donor 5 (**B**). #, DNA ladder; §, fibroblasts (control).

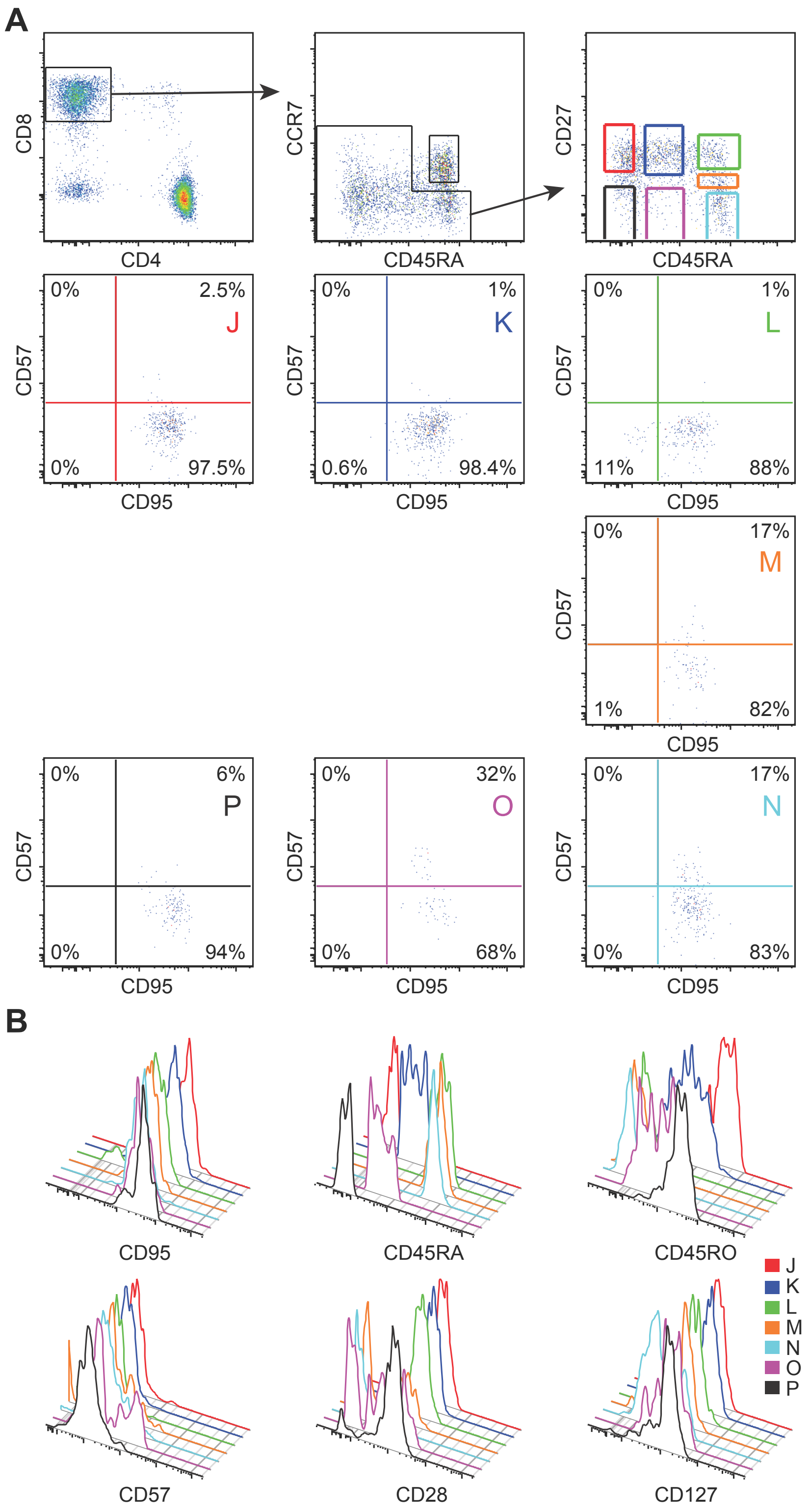

Supplementary Figure 2. Expression patterns of CD28, CD45RA, CD45RO, CD57, CD95, and CD127 among parental memory CD8^{+} T cell subsets flow-sorted for STELA. (**A**) Flow cytometric gating strategy for the isolation of subsets defined among populations J–P according to the expression of CD57 and CD95. (**B**) Histogram overlays showing the expression patterns of CD28, CD45RA, CD45RO, CD57, CD95, and CD127 among populations J–P. Data from donor 4.

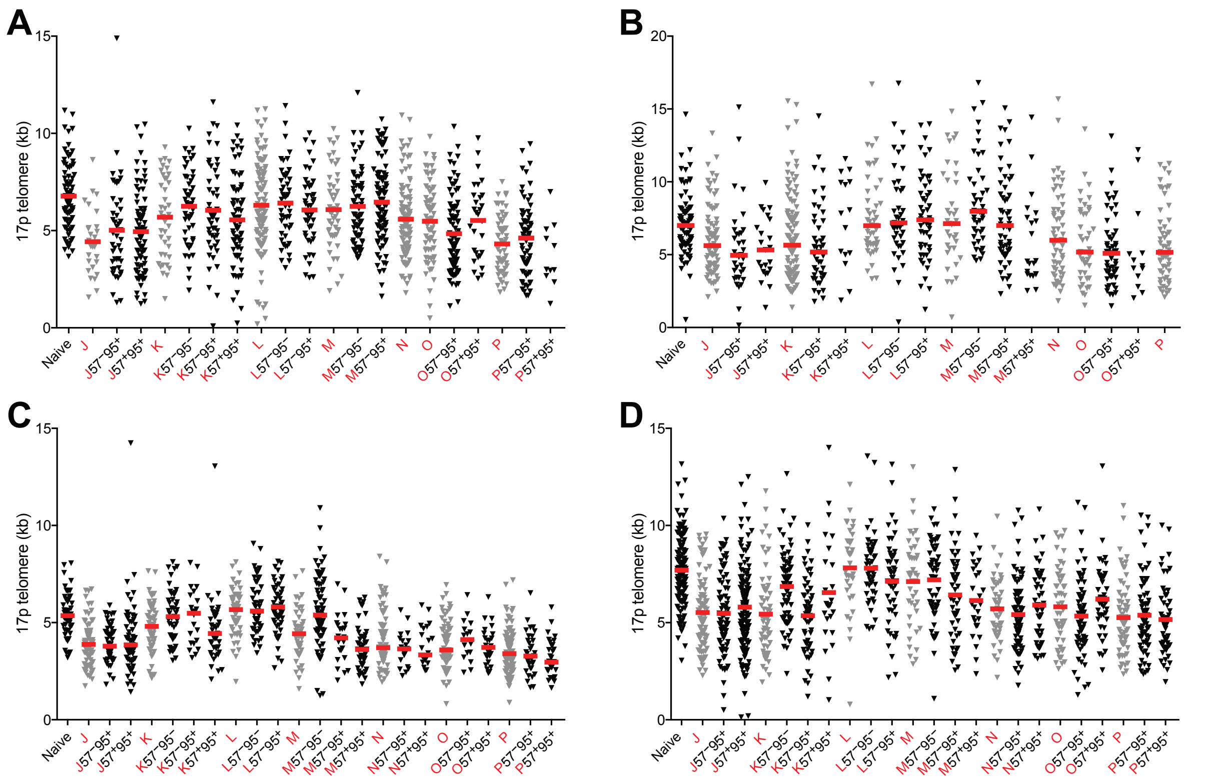

Supplementary Figure 3. Individual telomere length distribution patterns among CD8$^+$ memory T cell subsets stratified according to the expression of CD57 and CD95. (**A–D**) Scatter plots depicting 17p telomere length distributions from donors 1 (**A**), 3 (**B**), 4 (**C**), and 5 (**D**). Horizontal red lines indicate median values. Statistical comparisons across all sorted CD8$^+$ memory T cell subsets are shown in Supplementary Table 1. Donors 4 and 5 were known to be seropositive for CMV.

SUPPLEMENTARY TABLE

Supplementary Table 1. Telomere length data analyzed across all sorted $CD8^{+}$ memory T cell subsets using Dunn's *post-hoc* test for multiple comparisons.

T cell subsets	Mean rank difference	Adjusted p value
**Donor 1**		
Naive vs. J	425.6	**<0.0001**
Naive vs. $J57^{-}95^{+}$	332.4	**0.0003**
Naive vs. $J57^{+}95^{+}$	330.9	**<0.0001**
Naive vs. K	189.7	>0.9999
Naive vs. $K57^{-}95^{-}$	86.07	>0.9999
Naive vs. $K57^{-}95^{+}$	141.2	>0.9999
Naive vs. $K57^{+}95^{+}$	217.2	0.0630
Naive vs. L	82.98	>0.9999
Naive vs. $L57^{-}95^{-}$	68.21	>0.9999
Naive vs. $L57^{-}95^{+}$	123.1	>0.9999
Naive vs. M	128.5	>0.9999
Naive vs. $M57^{-}95^{-}$	103	>0.9999
Naive vs. $M57^{-}95^{+}$	62.14	>0.9999
Naive vs. N	223.2	**0.0124**
Naive vs. O	222.8	**0.0491**
Naive vs. $O57^{-}95^{+}$	352.9	**<0.0001**
Naive vs. $O57^{+}95^{+}$	222	>0.9999
Naive vs. P	445.7	**<0.0001**
Naive vs. $P57^{-}95^{+}$	388.3	**<0.0001**
Naive vs. $P57^{+}95^{+}$	537.3	**0.0012**
J vs. $J57^{-}95^{+}$	−93.26	>0.9999
J vs. $J57^{+}95^{+}$	−94.7	>0.9999
J vs. K	−235.9	>0.9999
J vs. $K57^{-}95^{-}$	−339.6	**0.0196**
J vs. $K57^{-}95^{+}$	−284.5	0.2143
J vs. $K57^{+}95^{+}$	−208.4	>0.9999
J vs. L	−342.7	**0.0025**
J vs. $L57^{-}95^{-}$	−357.4	**0.0094**
J vs. $L57^{-}95^{+}$	−302.5	0.1348
J vs. M	−297.2	0.1447
J vs. $M57^{-}95^{-}$	−322.6	**0.0193**
J vs. $M57^{-}95^{+}$	−363.5	**0.0010**
J vs. N	−202.5	>0.9999
J vs. O	−202.8	>0.9999
J vs. $O57^{-}95^{+}$	−72.78	>0.9999
J vs. $O57^{+}95^{+}$	−203.6	>0.9999
J vs. P	20.08	>0.9999
J vs. $P57^{-}95^{+}$	−37.33	>0.9999
J vs. $P57^{+}95^{+}$	111.7	>0.9999
$J57^{-}95^{+}$ vs. $J57^{+}95^{+}$	−1.442	>0.9999
$J57^{-}95^{+}$ vs. K	−142.7	>0.9999
$J57^{-}95^{+}$ vs. $K57^{-}95^{-}$	−246.3	0.2377
$J57^{-}95^{+}$ vs. $K57^{-}95^{+}$	−191.2	>0.9999
$J57^{-}95^{+}$ vs. $K57^{+}95^{+}$	−115.1	>0.9999

$J57^{-}95^{+}$ vs. L	−249.4	**0.0302**
$J57^{-}95^{+}$ vs. $L57^{-}95^{-}$	−264.2	0.1145
$J57^{-}95^{+}$ vs. $L57^{-}95^{+}$	−209.2	>0.9999
$J57^{-}95^{+}$ vs. M	−203.9	>0.9999
$J57^{-}95^{+}$ vs. $M57^{-}95^{-}$	−229.4	0.2412
$J57^{-}95^{+}$ vs. $M57^{-}95^{+}$	−270.2	**0.0118**
$J57^{-}95^{+}$ vs. N	−109.2	>0.9999
$J57^{-}95^{+}$ vs. O	−109.5	>0.9999
$J57^{-}95^{+}$ vs. $O57^{-}95^{+}$	20.48	>0.9999
$J57^{-}95^{+}$ vs. $O57^{+}95^{+}$	−110.4	>0.9999
$J57^{-}95^{+}$ vs. P	113.3	>0.9999
$J57^{-}95^{+}$ vs. $P57^{-}95^{+}$	55.93	>0.9999
$J57^{-}95^{+}$ vs. $P57^{+}95^{+}$	205	>0.9999
$J57^{+}95^{+}$ vs. K	−141.2	>0.9999
$J57^{+}95^{+}$ vs. $K57^{-}95^{-}$	−244.9	**0.0400**
$J57^{+}95^{+}$ vs. $K57^{-}95^{+}$	−189.8	0.7594
$J57^{+}95^{+}$ vs. $K57^{+}95^{+}$	−113.7	>0.9999
$J57^{+}95^{+}$ vs. L	−248	**0.0008**
$J57^{+}95^{+}$ vs. $L57^{-}95^{-}$	−262.7	**0.0163**
$J57^{+}95^{+}$ vs. $L57^{-}95^{+}$	−207.8	0.4628
$J57^{+}95^{+}$ vs. M	−202.5	0.4878
$J57^{+}95^{+}$ vs. $M57^{-}95^{-}$	−227.9	**0.0280**
$J57^{+}95^{+}$ vs. $M57^{-}95^{+}$	−268.8	**0.0003**
$J57^{+}95^{+}$ vs. N	−107.8	>0.9999
$J57^{+}95^{+}$ vs. O	−108.1	>0.9999
$J57^{+}95^{+}$ vs. $O57^{-}95^{+}$	21.92	>0.9999
$J57^{+}95^{+}$ vs. $O57^{+}95^{+}$	−108.9	>0.9999
$J57^{+}95^{+}$ vs. P	114.8	>0.9999
$J57^{+}95^{+}$ vs. $P57^{-}95^{+}$	57.38	>0.9999
$J57^{+}95^{+}$ vs. $P57^{+}95^{+}$	206.4	>0.9999
K vs. $K57^{-}95^{-}$	−103.6	>0.9999
K vs. $K57^{-}95^{+}$	−48.54	>0.9999
K vs. $K57^{+}95^{+}$	27.53	>0.9999
K vs. L	−106.7	>0.9999
K vs. $L57^{-}95^{-}$	−121.5	>0.9999
K vs. $L57^{-}95^{+}$	−66.57	>0.9999
K vs. M	−61.23	>0.9999
K vs. $M57^{-}95^{-}$	−86.72	>0.9999
K vs. $M57^{-}95^{+}$	−127.6	>0.9999
K vs. N	33.46	>0.9999
K vs. O	33.12	>0.9999
K vs. $O57^{-}95^{+}$	163.1	>0.9999
K vs. $O57^{+}95^{+}$	32.32	>0.9999
K vs. P	256	0.1580
K vs. $P57^{-}95^{+}$	198.6	>0.9999
K vs. $P57^{+}95^{+}$	347.6	>0.9999
$K57^{-}95^{-}$ vs. $K57^{-}95^{+}$	55.09	>0.9999
$K57^{-}95^{-}$ vs. $K57^{+}95^{+}$	131.2	>0.9999
$K57^{-}95^{-}$ vs. L	−3.085	>0.9999
$K57^{-}95^{-}$ vs. $L57^{-}95^{-}$	−17.86	>0.9999
$K57^{-}95^{-}$ vs. $L57^{-}95^{+}$	37.06	>0.9999
$K57^{-}95^{-}$ vs. M	42.4	>0.9999
$K57^{-}95^{-}$ vs. $M57^{-}95^{-}$	16.92	>0.9999

K57^{-}95^{-} vs. M57^{-}95^{+}	−23.93	>0.9999
K57^{-}95^{-} vs. N	137.1	>0.9999
K57^{-}95^{-} vs. O	136.8	>0.9999
K57^{-}95^{-} vs. O57^{-}95^{+}	266.8	**0.0093**
K57^{-}95^{-} vs. O57^{+}95^{+}	136	>0.9999
K57^{-}95^{-} vs. P	359.6	**0.0001**
K57^{-}95^{-} vs. P57^{-}95^{+}	302.2	**0.0017**
K57^{-}95^{-} vs. P57^{+}95^{+}	451.3	**0.0489**
K57^{-}95^{+} vs. K57^{+}95^{+}	76.08	>0.9999
K57^{-}95^{+} vs. L	−58.18	>0.9999
K57^{-}95^{+} vs. L57^{-}95^{-}	−72.95	>0.9999
K57^{-}95^{+} vs. L57^{-}95^{+}	−18.03	>0.9999
K57^{-}95^{+} vs. M	−12.69	>0.9999
K57^{-}95^{+} vs. M57^{-}95^{-}	−38.17	>0.9999
K57^{-}95^{+} vs. M57^{-}95^{+}	−79.02	>0.9999
K57^{-}95^{+} vs. N	82.01	>0.9999
K57^{-}95^{+} vs. O	81.67	>0.9999
K57^{-}95^{+} vs. O57^{-}95^{+}	211.7	0.2340
K57^{-}95^{+} vs. O57^{+}95^{+}	80.86	>0.9999
K57^{-}95^{+} vs. P	304.6	**0.0044**
K57^{-}95^{+} vs. P57^{-}95^{+}	247.1	0.0514
K57^{-}95^{+} vs. P57^{+}95^{+}	396.2	0.2540
K57^{+}95^{+} vs. L	−134.3	>0.9999
K57^{+}95^{+} vs. L57^{-}95^{-}	−149	>0.9999
K57^{+}95^{+} vs. L57^{-}95^{+}	−94.11	>0.9999
K57^{+}95^{+} vs. M	−88.77	>0.9999
K57^{+}95^{+} vs. M57^{-}95^{-}	−114.2	>0.9999
K57^{+}95^{+} vs. M57^{-}95^{+}	−155.1	>0.9999
K57^{+}95^{+} vs. N	5.931	>0.9999
K57^{+}95^{+} vs. O	5.591	>0.9999
K57^{+}95^{+} vs. O57^{-}95^{+}	135.6	>0.9999
K57^{+}95^{+} vs. O57^{+}95^{+}	4.788	>0.9999
K57^{+}95^{+} vs. P	228.5	0.1205
K57^{+}95^{+} vs. P57^{-}95^{+}	171.1	>0.9999
K57^{+}95^{+} vs. P57^{+}95^{+}	320.1	>0.9999
L vs. L57^{-}95^{-}	−14.77	>0.9999
L vs. L57^{-}95^{+}	40.15	>0.9999
L vs. M	45.49	>0.9999
L vs. M57^{-}95^{-}	20.01	>0.9999
L vs. M57^{-}95^{+}	−20.84	>0.9999
L vs. N	140.2	>0.9999
L vs. O	139.8	>0.9999
L vs. O57^{-}95^{+}	269.9	**<0.0001**
L vs. O57^{+}95^{+}	139	>0.9999
L vs. P	362.7	**<0.0001**
L vs. P57^{-}95^{+}	305.3	**<0.0001**
L vs. P57^{+}95^{+}	454.4	**0.0207**
L57^{-}95^{-} vs. L57^{-}95^{+}	54.92	>0.9999
L57^{-}95^{-} vs. M	60.26	>0.9999
L57^{-}95^{-} vs. M57^{-}95^{-}	34.78	>0.9999
L57^{-}95^{-} vs. M57^{-}95^{+}	−6.07	>0.9999
L57^{-}95^{-} vs. N	155	>0.9999
L57^{-}95^{-} vs. O	154.6	>0.9999

L$57^{-}95^{-}$ vs. O$57^{-}95^{+}$	284.6	**0.0036**
L$57^{-}95^{-}$ vs. O$57^{+}95^{+}$	153.8	>0.9999
L$57^{-}95^{-}$ vs. P	377.5	**<0.0001**
L$57^{-}95^{-}$ vs. P$57^{-}95^{+}$	320.1	**0.0006**
L$57^{-}95^{-}$ vs. P$57^{+}95^{+}$	469.1	**0.0290**
L$57^{-}95^{+}$ vs. M	5.339	>0.9999
L$57^{-}95^{+}$ vs. M$57^{-}95^{-}$	−20.14	>0.9999
L$57^{-}95^{+}$ vs. M$57^{-}95^{+}$	−60.99	>0.9999
L$57^{-}95^{+}$ vs. N	100	>0.9999
L$57^{-}95^{+}$ vs. O	99.7	>0.9999
L$57^{-}95^{+}$ vs. O$57^{-}95^{+}$	229.7	0.1427
L$57^{-}95^{+}$ vs. O$57^{+}95^{+}$	98.89	>0.9999
L$57^{-}95^{+}$ vs. P	322.6	**0.0027**
L$57^{-}95^{+}$ vs. P$57^{-}95^{+}$	265.2	**0.0316**
L$57^{-}95^{+}$ vs. P$57^{+}95^{+}$	414.2	0.1731
M vs. M$57^{-}95^{-}$	−25.48	>0.9999
M vs. M$57^{-}95^{+}$	−66.33	>0.9999
M vs. N	94.7	>0.9999
M vs. O	94.36	>0.9999
M vs. O$57^{-}95^{+}$	224.4	0.1471
M vs. O$57^{+}95^{+}$	93.55	>0.9999
M vs. P	317.2	**0.0027**
M vs. P$57^{-}95^{+}$	259.8	**0.0320**
M vs. P$57^{+}95^{+}$	408.9	0.1877
M$57^{-}95^{-}$ vs. M$57^{-}95^{+}$	−40.85	>0.9999
M$57^{-}95^{-}$ vs. N	120.2	>0.9999
M$57^{-}95^{-}$ vs. O	119.8	>0.9999
M$57^{-}95^{-}$ vs. O$57^{-}95^{+}$	249.9	**0.0054**
M$57^{-}95^{-}$ vs. O$57^{+}95^{+}$	119	>0.9999
M$57^{-}95^{-}$ vs. P	342.7	**<0.0001**
M$57^{-}95^{-}$ vs. P$57^{-}95^{+}$	285.3	**0.0009**
M$57^{-}95^{-}$ vs. P$57^{+}95^{+}$	434.4	0.0587
M$57^{-}95^{+}$ vs. N	161	0.5875
M$57^{-}95^{+}$ vs. O	160.7	>0.9999
M$57^{-}95^{+}$ vs. O$57^{-}95^{+}$	290.7	**<0.0001**
M$57^{-}95^{+}$ vs. O$57^{+}95^{+}$	159.9	>0.9999
M$57^{-}95^{+}$ vs. P	383.6	**<0.0001**
M$57^{-}95^{+}$ vs. P$57^{-}95^{+}$	326.2	**<0.0001**
M$57^{-}95^{+}$ vs. P$57^{+}95^{+}$	475.2	**0.0111**
O vs. N	0.3401	>0.9999
O vs. O$57^{-}95^{+}$	130	>0.9999
O vs. O$57^{+}95^{+}$	−0.8032	>0.9999
O vs. P	222.9	0.1773
O vs. P$57^{-}95^{+}$	165.5	>0.9999
O vs. P$57^{+}95^{+}$	314.5	>0.9999
O$57^{-}95^{+}$ vs. N	−129.7	>0.9999
O$57^{-}95^{+}$ vs. O$57^{+}95^{+}$	−130.8	>0.9999
O$57^{-}95^{+}$ vs. P	92.86	>0.9999
O$57^{-}95^{+}$ vs. P$57^{-}95^{+}$	35.46	>0.9999
O$57^{-}95^{+}$ vs. P$57^{+}95^{+}$	184.5	>0.9999
O$57^{+}95^{+}$ vs. N	1.143	>0.9999
O$57^{+}95^{+}$ vs. P	223.7	>0.9999
O$57^{+}95^{+}$ vs. P$57^{-}95^{+}$	166.3	>0.9999

O57+95+ vs. P57+95+	315.3	>0.9999
P vs. N	−222.5	0.0738
P vs. P57‾95+	−57.4	>0.9999
P vs. P57+95+	91.63	>0.9999
P57‾95+ vs. N	−165.1	0.8435
P57‾95+ vs. P57+95+	149	>0.9999
P57+95+ vs. N	−314.2	>0.9999
**Donor 3**		
Naive vs. J	127	0.3772
Naive vs. J57‾95+	202.1	**0.0240**
Naive vs. J57+95+	178.3	0.6763
Naive vs. K	120.1	0.3475
Naive vs. K57‾95+	172.4	0.0706
Naive vs. K57+95+	14.97	>0.9999
Naive vs. L	−13.98	>0.9999
Naive vs. L57‾95‾	−28.03	>0.9999
Naive vs. L57‾95+	−11.77	>0.9999
Naive vs. M	−3.981	>0.9999
Naive vs. M57‾95‾	−89.1	>0.9999
Naive vs. M57‾95+	9.882	>0.9999
Naive vs. M57+95+	162.6	>0.9999
Naive vs. N	98.47	>0.9999
Naive vs. O	156.9	0.2864
Naive vs. O57‾95+	204.8	**0.0013**
Naive vs. O57+95+	230.2	0.5505
Naive vs. P	166.7	**0.0450**
J vs. J57‾95+	75.01	>0.9999
J vs. J57+95+	51.3	>0.9999
J vs. K	−6.99	>0.9999
J vs. K57‾95+	45.4	>0.9999
J vs. K57+95+	−112.1	>0.9999
J vs. L	−141	0.5578
J vs. L57‾95‾	−155.1	0.3359
J vs. L57‾95+	−138.8	0.5089
J vs. M	−131	>0.9999
J vs. M57‾95‾	−216.1	**0.0015**
J vs. M57‾95+	−117.2	>0.9999
J vs. M57+95+	35.55	>0.9999
J vs. N	−28.57	>0.9999
J vs. O	29.83	>0.9999
J vs. O57‾95+	77.76	>0.9999
J vs. O57+95+	103.1	>0.9999
J vs. P	39.66	>0.9999
J57‾95+ vs. J57+95+	−23.72	>0.9999
J57‾95+ vs. K	−82	>0.9999
J57‾95+ vs. K57‾95+	−29.61	>0.9999
J57‾95+ vs. K57+95+	−187.1	>0.9999
J57‾95+ vs. L	−216	**0.0355**
J57‾95+ vs. L57‾95‾	−230.1	**0.0216**
J57‾95+ vs. L57‾95+	−213.8	**0.0321**
J57‾95+ vs. M	−206	0.1381
J57‾95+ vs. M57‾95‾	−291.2	**0.0001**
J57‾95+ vs. M57‾95+	−192.2	0.1172

J57$^-$95$^+$ vs. M57$^+$95$^+$	−39.46	>0.9999
J57$^-$95$^+$ vs. N	−103.6	>0.9999
J57$^-$95$^+$ vs. O	−45.19	>0.9999
J57$^-$95$^+$ vs. O57$^-$95$^+$	2.746	>0.9999
J57$^-$95$^+$ vs. O57$^+$95$^+$	28.12	>0.9999
J57$^-$95$^+$ vs. P	−35.35	>0.9999
J57$^+$95$^+$ vs. K	−58.29	>0.9999
J57$^+$95$^+$ vs. K57$^-$95$^+$	−5.896	>0.9999
J57$^+$95$^+$ vs. K57$^+$95$^+$	−163.4	>0.9999
J57$^+$95$^+$ vs. L	−192.3	0.6431
J57$^+$95$^+$ vs. L57$^-$95$^-$	−206.4	0.4084
J57$^+$95$^+$ vs. L57$^-$95$^+$	−190.1	0.6341
J57$^+$95$^+$ vs. M	−182.3	>0.9999
J57$^+$95$^+$ vs. M57$^-$95$^-$	−267.4	**0.0108**
J57$^+$95$^+$ vs. M57$^-$95$^+$	−168.5	>0.9999
J57$^+$95$^+$ vs. M57$^+$95$^+$	−15.74	>0.9999
J57$^+$95$^+$ vs. N	−79.87	>0.9999
J57$^+$95$^+$ vs. O	−21.47	>0.9999
J57$^+$95$^+$ vs. O57$^-$95$^+$	26.47	>0.9999
J57$^+$95$^+$ vs. O57$^+$95$^+$	51.84	>0.9999
J57$^+$95$^+$ vs. P	−11.63	>0.9999
K vs. K57$^-$95$^+$	52.39	>0.9999
K vs. K57$^+$95$^+$	−105.1	>0.9999
K vs. L	−134	0.5753
K vs. L57$^-$95$^-$	−148.1	0.3456
K vs. L57$^-$95$^+$	−131.8	0.5163
K vs. M	−124	>0.9999
K vs. M57$^-$95$^-$	−209.2	**0.0011**
K vs. M57$^-$95$^+$	−110.2	>0.9999
K vs. M57$^+$95$^+$	42.54	>0.9999
K vs. N	−21.58	>0.9999
K vs. O	36.82	>0.9999
K vs. O57$^-$95$^+$	84.75	>0.9999
K vs. O57$^+$95$^+$	110.1	>0.9999
K vs. P	46.65	>0.9999
K57$^-$95$^+$ vs. K57$^+$95$^+$	−157.5	>0.9999
K57$^-$95$^+$ vs. L	−186.4	0.1044
K57$^-$95$^+$ vs. L57$^-$95$^-$	−200.5	0.0634
K57$^-$95$^+$ vs. L57$^-$95$^+$	−184.2	0.0947
K57$^-$95$^+$ vs. M	−176.4	0.3939
K57$^-$95$^+$ vs. M57$^-$95$^-$	−261.5	**0.0003**
K57$^-$95$^+$ vs. M57$^-$95$^+$	−162.6	0.3447
K57$^-$95$^+$ vs. M57$^+$95$^+$	−9.848	>0.9999
K57$^-$95$^+$ vs. N	−73.97	>0.9999
K57$^-$95$^+$ vs. O	−15.57	>0.9999
K57$^-$95$^+$ vs. O57$^-$95$^+$	32.36	>0.9999
K57$^-$95$^+$ vs. O57$^+$95$^+$	57.74	>0.9999
K57$^-$95$^+$ vs. P	−5.738	>0.9999
K57$^+$95$^+$ vs. L	−28.95	>0.9999
K57$^+$95$^+$ vs. L57$^-$95$^-$	−43	>0.9999
K57$^+$95$^+$ vs. L57$^-$95$^+$	−26.75	>0.9999
K57$^+$95$^+$ vs. M	−18.95	>0.9999
K57$^+$95$^+$ vs. M57$^-$95$^-$	−104.1	>0.9999

K57^{+}95^{+} vs. M57^{-}95^{+}	−5.093	>0.9999
K57^{+}95^{+} vs. M57^{+}95^{+}	147.6	>0.9999
K57^{+}95^{+} vs. N	83.49	>0.9999
K57^{+}95^{+} vs. O	141.9	>0.9999
K57^{+}95^{+} vs. O57^{-}95^{+}	189.8	>0.9999
K57^{+}95^{+} vs. O57^{+}95^{+}	215.2	>0.9999
K57^{+}95^{+} vs. P	151.7	>0.9999
L vs. L57^{-}95^{-}	−14.05	>0.9999
L vs. L57^{-}95^{+}	2.205	>0.9999
L vs. M	9.995	>0.9999
L vs. M57^{-}95^{-}	−75.13	>0.9999
L vs. M57^{-}95^{+}	23.86	>0.9999
L vs. M57^{+}95^{+}	176.6	>0.9999
L vs. N	112.4	>0.9999
L vs. O	170.8	0.3482
L vs. O57^{-}95^{+}	218.8	**0.0038**
L vs. O57^{+}95^{+}	244.2	0.4813
L vs. P	180.7	0.0790
L57^{-}95^{-} vs. L57^{-}95^{+}	16.26	>0.9999
L57^{-}95^{-} vs. M	24.05	>0.9999
L57^{-}95^{-} vs. M57^{-}95^{-}	−61.07	>0.9999
L57^{-}95^{-} vs. M57^{-}95^{+}	37.91	>0.9999
L57^{-}95^{-} vs. M57^{+}95^{+}	190.6	>0.9999
L57^{-}95^{-} vs. N	126.5	>0.9999
L57^{-}95^{-} vs. O	184.9	0.2124
L57^{-}95^{-} vs. O57^{-}95^{+}	232.8	**0.0024**
L57^{-}95^{-} vs. O57^{+}95^{+}	258.2	0.3196
L57^{-}95^{-} vs. P	194.7	**0.0480**
L57^{-}95^{+} vs. M	7.79	>0.9999
L57^{-}95^{+} vs. M57^{-}95^{-}	−77.33	>0.9999
L57^{-}95^{+} vs. M57^{-}95^{+}	21.65	>0.9999
L57^{-}95^{+} vs. M57^{+}95^{+}	174.4	>0.9999
L57^{-}95^{+} vs. N	110.2	>0.9999
L57^{-}95^{+} vs. O	168.6	0.3275
L57^{-}95^{+} vs. O57^{-}95^{+}	216.6	**0.0031**
L57^{-}95^{+} vs. O57^{+}95^{+}	242	0.4837
L57^{-}95^{+} vs. P	178.5	0.0697
M vs. M57^{-}95^{-}	−85.12	>0.9999
M vs. M57^{-}95^{+}	13.86	>0.9999
M vs. M57^{+}95^{+}	166.6	>0.9999
M vs. N	102.4	>0.9999
M vs. O	160.8	>0.9999
M vs. O57^{-}95^{+}	208.8	**0.0270**
M vs. O57^{+}95^{+}	234.2	0.9164
M vs. P	170.7	0.3435
M57^{-}95^{-} vs. M57^{-}95^{+}	98.99	>0.9999
M57^{-}95^{-} vs. M57^{+}95^{+}	251.7	**0.0446**
M57^{-}95^{-} vs. N	187.6	0.0503
M57^{-}95^{-} vs. O	246	**0.0019**
M57^{-}95^{-} vs. O57^{-}95^{+}	293.9	**<0.0001**
M57^{-}95^{-} vs. O57^{+}95^{+}	319.3	**0.0173**
M57^{-}95^{-} vs. P	255.8	**0.0002**
M57^{-}95^{+} vs. M57^{+}95^{+}	152.7	>0.9999

M$57^{-}95^{+}$ vs. N	88.59	>0.9999
M$57^{-}95^{+}$ vs. O	147	>0.9999
M$57^{-}95^{+}$ vs. O$57^{-}95^{+}$	194.9	**0.0152**
M$57^{-}95^{+}$ vs. O$57^{+}95^{+}$	220.3	>0.9999
M$57^{-}95^{+}$ vs. P	156.8	0.2768
M$57^{+}95^{+}$ vs. N	−64.12	>0.9999
M$57^{+}95^{+}$ vs. O	−5.726	>0.9999
M$57^{+}95^{+}$ vs. O$57^{-}95^{+}$	42.21	>0.9999
M$57^{+}95^{+}$ vs. O$57^{+}95^{+}$	67.59	>0.9999
M$57^{+}95^{+}$ vs. P	4.11	>0.9999
N vs. O	58.4	>0.9999
N vs. O$57^{-}95^{+}$	106.3	>0.9999
N vs. O$57^{+}95^{+}$	131.7	>0.9999
N vs. P	68.23	>0.9999
O vs. O$57^{-}95^{+}$	47.93	>0.9999
O vs. O$57^{+}95^{+}$	73.31	>0.9999
O vs. P	9.836	>0.9999
O$57^{-}95^{+}$ vs. O$57^{+}95^{+}$	25.38	>0.9999
O$57^{-}95^{+}$ vs. P	−38.1	>0.9999
O$57^{+}95^{+}$ vs. P	−63.48	>0.9999
**Donor 4**		
Naive vs. J	333.6	**<0.0001**
Naive vs. J$57^{-}95^{+}$	332.2	**<0.0001**
Naive vs. J$57^{+}95^{+}$	302.7	**0.0002**
Naive vs. K	135.4	>0.9999
Naive vs. K$57^{-}95^{-}$	38.08	>0.9999
Naive vs. K$57^{-}95^{+}$	50.82	>0.9999
Naive vs. K$57^{+}95^{+}$	204.8	0.7202
Naive vs. L	−23.46	>0.9999
Naive vs. L$57^{-}95^{-}$	−53.94	>0.9999
Naive vs. L$57^{-}95^{+}$	−51.7	>0.9999
Naive vs. M	218.9	0.2606
Naive vs. M$57^{-}95^{-}$	6.985	>0.9999
Naive vs. M$57^{-}95^{+}$	273	0.1575
Naive vs. M$57^{+}95^{+}$	376	**<0.0001**
Naive vs. N	308.2	**<0.0001**
Naive vs. N$57^{-}95^{+}$	406.3	**0.0003**
Naive vs. N$57^{+}95^{+}$	377.9	**0.0010**
Naive vs. O	337.5	**<0.0001**
Naive vs. O$57^{-}95^{+}$	293.6	0.3816
Naive vs. O$57^{+}95^{+}$	367.4	**0.0005**
Naive vs. P	429.3	**<0.0001**
Naive vs. P$57^{-}95^{+}$	457.2	**<0.0001**
Naive vs. P$57^{+}95^{+}$	512.9	**<0.0001**
J vs. J$57^{-}95^{+}$	−1.407	>0.9999
J vs. J$57^{+}95^{+}$	−30.97	>0.9999
J vs. K	−198.2	0.1102
J vs. K$57^{-}95^{-}$	−295.6	**0.0003**
J vs. K$57^{-}95^{+}$	−282.8	0.0759
J vs. K$57^{+}95^{+}$	−128.9	>0.9999
J vs. L	−357.1	**<0.0001**
J vs. L$57^{-}95^{-}$	−387.6	**<0.0001**
J vs. L$57^{-}95^{+}$	−385.3	**<0.0001**

J vs. M	−114.8	>0.9999
J vs. M57^{-}95^{-}	−326.7	**<0.0001**
J vs. M57^{-}95^{+}	−60.61	>0.9999
J vs. M57^{+}95^{+}	42.39	>0.9999
J vs. N	−25.46	>0.9999
J vs. N57^{-}95^{+}	72.62	>0.9999
J vs. N57^{+}95^{+}	44.21	>0.9999
J vs. O	3.814	>0.9999
J vs. O57^{-}95^{+}	−40.01	>0.9999
J vs. O57^{+}95^{+}	33.74	>0.9999
J vs. P	95.68	>0.9999
J vs. P57^{-}95^{+}	123.6	>0.9999
J vs. P57^{+}95^{+}	179.3	>0.9999
J57^{-}95^{+} vs. J57^{+}95^{+}	−29.57	>0.9999
J57^{-}95^{+} vs. K	−196.8	0.4372
J57^{-}95^{+} vs. K57^{-}95^{-}	−294.2	**0.0025**
J57^{-}95^{+} vs. K57^{-}95^{+}	−281.4	0.1763
J57^{-}95^{+} vs. K57^{+}95^{+}	−127.5	>0.9999
J57^{-}95^{+} vs. L	−355.7	**<0.0001**
J57^{-}95^{+} vs. L57^{-}95^{-}	−386.2	**<0.0001**
J57^{-}95^{+} vs. L57^{-}95^{+}	−383.9	**<0.0001**
J57^{-}95^{+} vs. M	−113.4	>0.9999
J57^{-}95^{+} vs. M57^{-}95^{-}	−325.3	**<0.0001**
J57^{-}95^{+} vs. M57^{-}95^{+}	−59.21	>0.9999
J57^{-}95^{+} vs. M57^{+}95^{+}	43.8	>0.9999
J57^{-}95^{+} vs. N	−24.06	>0.9999
J57^{-}95^{+} vs. N57^{-}95^{+}	74.03	>0.9999
J57^{-}95^{+} vs. N57^{+}95^{+}	45.62	>0.9999
J57^{-}95^{+} vs. O	5.221	>0.9999
J57^{-}95^{+} vs. O57^{-}95^{+}	−38.6	>0.9999
J57^{-}95^{+} vs. O57^{+}95^{+}	35.15	>0.9999
J57^{-}95^{+} vs. P	97.08	>0.9999
J57^{-}95^{+} vs. P57^{-}95^{+}	125	>0.9999
J57^{-}95^{+} vs. P57^{+}95^{+}	180.7	>0.9999
J57^{+}95^{+} vs. K	−167.3	>0.9999
J57^{+}95^{+} vs. K57^{-}95^{-}	−264.6	**0.0099**
J57^{+}95^{+} vs. K57^{-}95^{+}	−251.9	0.4915
J57^{+}95^{+} vs. K57^{+}95^{+}	−97.88	>0.9999
J57^{+}95^{+} vs. L	−326.1	**<0.0001**
J57^{+}95^{+} vs. L57^{-}95^{-}	−356.6	**<0.0001**
J57^{+}95^{+} vs. L57^{-}95^{+}	−354.4	**<0.0001**
J57^{+}95^{+} vs. M	−83.79	>0.9999
J57^{+}95^{+} vs. M57^{-}95^{-}	−295.7	**<0.0001**
J57^{+}95^{+} vs. M57^{-}95^{+}	−29.64	>0.9999
J57^{+}95^{+} vs. M57^{+}95^{+}	73.36	>0.9999
J57^{+}95^{+} vs. N	5.512	>0.9999
J57^{+}95^{+} vs. N57^{-}95^{+}	103.6	>0.9999
J57^{+}95^{+} vs. N57^{+}95^{+}	75.19	>0.9999
J57^{+}95^{+} vs. O	34.79	>0.9999
J57^{+}95^{+} vs. O57^{-}95^{+}	−9.036	>0.9999
J57^{+}95^{+} vs. O57^{+}95^{+}	64.72	>0.9999
J57^{+}95^{+} vs. P	126.6	>0.9999
J57^{+}95^{+} vs. P57^{-}95^{+}	154.6	>0.9999

J57^{+}95^{+} vs. P57^{+}95^{+}	210.2	0.8389
K vs. K57^{-}95^{-}	−97.32	>0.9999
K vs. K57^{-}95^{+}	−84.58	>0.9999
K vs. K57^{+}95^{+}	69.39	>0.9999
K vs. L	−158.9	>0.9999
K vs. L57^{-}95^{-}	−189.3	0.7355
K vs. L57^{-}95^{+}	−187.1	0.6640
K vs. M	83.49	>0.9999
K vs. M57^{-}95^{-}	−128.4	>0.9999
K vs. M57^{-}95^{+}	137.6	>0.9999
K vs. M57^{+}95^{+}	240.6	0.0542
K vs. N	172.8	0.6747
K vs. N57^{-}95^{+}	270.9	0.2558
K vs. N57^{+}95^{+}	242.5	0.7088
K vs. O	202.1	0.1367
K vs. O57^{-}95^{+}	158.2	>0.9999
K vs. O57^{+}95^{+}	232	0.6093
K vs. P	293.9	**<0.0001**
K vs. P57^{-}95^{+}	321.8	**0.0012**
K vs. P57^{+}95^{+}	377.5	**<0.0001**
K57^{-}95^{-} vs. K57^{-}95^{+}	12.74	>0.9999
K57^{-}95^{-} vs. K57^{+}95^{+}	166.7	>0.9999
K57^{-}95^{-} vs. L	−61.54	>0.9999
K57^{-}95^{-} vs. L57^{-}95^{-}	−92.02	>0.9999
K57^{-}95^{-} vs. L57^{-}95^{+}	−89.78	>0.9999
K57^{-}95^{-} vs. M	180.8	>0.9999
K57^{-}95^{-} vs. M57^{-}95^{-}	−31.09	>0.9999
K57^{-}95^{-} vs. M57^{-}95^{+}	235	>0.9999
K57^{-}95^{-} vs. M57^{+}95^{+}	338	**0.0002**
K57^{-}95^{-} vs. N	270.1	**0.0030**
K57^{-}95^{-} vs. N57^{-}95^{+}	368.2	**0.0040**
K57^{-}95^{-} vs. N57^{+}95^{+}	339.8	**0.0131**
K57^{-}95^{-} vs. O	299.4	**0.0004**
K57^{-}95^{-} vs. O57^{-}95^{+}	255.6	>0.9999
K57^{-}95^{-} vs. O57^{+}95^{+}	329.3	**0.0087**
K57^{-}95^{-} vs. P	391.2	**<0.0001**
K57^{-}95^{-} vs. P57^{-}95^{+}	419.2	**<0.0001**
K57^{-}95^{-} vs. P57^{+}95^{+}	474.8	**<0.0001**
K57^{-}95^{+} vs. K57^{+}95^{+}	154	>0.9999
K57^{-}95^{+} vs. L	−74.28	>0.9999
K57^{-}95^{+} vs. L57^{-}95^{-}	−104.8	>0.9999
K57^{-}95^{+} vs. L57^{-}95^{+}	−102.5	>0.9999
K57^{-}95^{+} vs. M	168.1	>0.9999
K57^{-}95^{+} vs. M57^{-}95^{-}	−43.83	>0.9999
K57^{-}95^{+} vs. M57^{-}95^{+}	222.2	>0.9999
K57^{-}95^{+} vs. M57^{+}95^{+}	325.2	**0.0309**
K57^{-}95^{+} vs. N	257.4	0.2877
K57^{-}95^{+} vs. N57^{-}95^{+}	355.5	0.0788
K57^{-}95^{+} vs. N57^{+}95^{+}	327	0.2019
K57^{-}95^{+} vs. O	286.6	0.0817
K57^{-}95^{+} vs. O57^{-}95^{+}	242.8	>0.9999
K57^{-}95^{+} vs. O57^{+}95^{+}	316.6	0.1840
K57^{-}95^{+} vs. P	378.5	**0.0002**

K57^{-}95^{+} vs. P57^{-}95^{+}	406.4	**0.0012**
K57^{-}95^{+} vs. P57^{+}95^{+}	462.1	**<0.0001**
K57^{+}95^{+} vs. L	−228.3	0.2189
K57^{+}95^{+} vs. L57^{-}95^{-}	−258.7	0.0551
K57^{+}95^{+} vs. L57^{-}95^{+}	−256.5	**0.0479**
K57^{+}95^{+} vs. M	14.1	>0.9999
K57^{+}95^{+} vs. M57^{-}95^{-}	−197.8	0.4875
K57^{+}95^{+} vs. M57^{-}95^{+}	68.24	>0.9999
K57^{+}95^{+} vs. M57^{+}95^{+}	171.2	>0.9999
K57^{+}95^{+} vs. N	103.4	>0.9999
K57^{+}95^{+} vs. N57^{-}95^{+}	201.5	>0.9999
K57^{+}95^{+} vs. N57^{+}95^{+}	173.1	>0.9999
K57^{+}95^{+} vs. O	132.7	>0.9999
K57^{+}95^{+} vs. O57^{-}95^{+}	88.85	>0.9999
K57^{+}95^{+} vs. O57^{+}95^{+}	162.6	>0.9999
K57^{+}95^{+} vs. P	224.5	0.0683
K57^{+}95^{+} vs. P57^{-}95^{+}	252.5	0.2453
K57^{+}95^{+} vs. P57^{+}95^{+}	308.1	**0.0159**
L vs. L57^{-}95^{-}	−30.47	>0.9999
L vs. L57^{-}95^{+}	−28.24	>0.9999
L vs. M	242.3	0.0693
L vs. M57^{-}95^{-}	30.45	>0.9999
L vs. M57^{-}95^{+}	296.5	0.0505
L vs. M57^{+}95^{+}	399.5	**<0.0001**
L vs. N	331.6	**<0.0001**
L vs. N57^{-}95^{+}	429.7	**<0.0001**
L vs. N57^{+}95^{+}	401.3	**0.0002**
L vs. O	360.9	**<0.0001**
L vs. O57^{-}95^{+}	317.1	0.1526
L vs. O57^{+}95^{+}	390.9	**0.0001**
L vs. P	452.8	**<0.0001**
L vs. P57^{-}95^{+}	480.7	**<0.0001**
L vs. P57^{+}95^{+}	536.4	**<0.0001**
L57^{-}95^{-} vs. L57^{-}95^{+}	2.236	>0.9999
L57^{-}95^{-} vs. M	272.8	**0.0157**
L57^{-}95^{-} vs. M57^{-}95^{-}	60.92	>0.9999
L57^{-}95^{-} vs. M57^{-}95^{+}	327	**0.0137**
L57^{-}95^{-} vs. M57^{+}95^{+}	430	**<0.0001**
L57^{-}95^{-} vs. N	362.1	**<0.0001**
L57^{-}95^{-} vs. N57^{-}95^{+}	460.2	**<0.0001**
L57^{-}95^{-} vs. N57^{+}95^{+}	431.8	**<0.0001**
L57^{-}95^{-} vs. O	391.4	**<0.0001**
L57^{-}95^{-} vs. O57^{-}95^{+}	347.6	0.0510
L57^{-}95^{-} vs. O57^{+}95^{+}	421.3	**<0.0001**
L57^{-}95^{-} vs. P	483.3	**<0.0001**
L57^{-}95^{-} vs. P57^{-}95^{+}	511.2	**<0.0001**
L57^{-}95^{-} vs. P57^{+}95^{+}	566.9	**<0.0001**
L57^{-}95^{+} vs. M	270.6	**0.0130**
L57^{-}95^{+} vs. M57^{-}95^{-}	58.68	>0.9999
L57^{-}95^{+} vs. M57^{-}95^{+}	324.7	**0.0122**
L57^{-}95^{+} vs. M57^{+}95^{+}	427.7	**<0.0001**
L57^{-}95^{+} vs. N	359.9	**<0.0001**
L57^{-}95^{+} vs. N57^{-}95^{+}	458	**<0.0001**

$L57^{-}95^{+}$ vs. $N57^{+}95^{+}$	429.6	**<0.0001**
$L57^{-}95^{+}$ vs. O	389.2	**<0.0001**
$L57^{-}95^{+}$ vs. $O57^{-}95^{+}$	345.3	**0.0483**
$L57^{-}95^{+}$ vs. $O57^{+}95^{+}$	419.1	**<0.0001**
$L57^{-}95^{+}$ vs. P	481	**<0.0001**
$L57^{-}95^{+}$ vs. $P57^{-}95^{+}$	508.9	**<0.0001**
$L57^{-}95^{+}$ vs. $P57^{+}95^{+}$	564.6	**<0.0001**
M vs. $M57^{-}95^{-}$	−211.9	0.1503
M vs. $M57^{-}95^{+}$	54.15	>0.9999
M vs. $M57^{+}95^{+}$	157.2	>0.9999
M vs. N	89.3	>0.9999
M vs. $N57^{-}95^{+}$	187.4	>0.9999
M vs. $N57^{+}95^{+}$	159	>0.9999
M vs. O	118.6	>0.9999
M vs. $O57^{-}95^{+}$	74.75	>0.9999
M vs. $O57^{+}95^{+}$	148.5	>0.9999
M vs. P	210.4	0.1050
M vs. $P57^{-}95^{+}$	238.4	0.3701
M vs. $P57^{+}95^{+}$	294	**0.0243**
$M57^{-}95^{-}$ vs. $M57^{-}95^{+}$	266.1	0.1112
$M57^{-}95^{-}$ vs. $M57^{+}95^{+}$	369.1	**<0.0001**
$M57^{-}95^{-}$ vs. N	301.2	**<0.0001**
$M57^{-}95^{-}$ vs. $N57^{-}95^{+}$	399.3	**0.0001**
$M57^{-}95^{-}$ vs. $N57^{+}95^{+}$	370.9	**0.0005**
$M57^{-}95^{-}$ vs. O	330.5	**<0.0001**
$M57^{-}95^{-}$ vs. $O57^{-}95^{+}$	286.7	0.3241
$M57^{-}95^{-}$ vs. $O57^{+}95^{+}$	360.4	**0.0002**
$M57^{-}95^{-}$ vs. P	422.3	**<0.0001**
$M57^{-}95^{-}$ vs. $P57^{-}95^{+}$	450.3	**<0.0001**
$M57^{-}95^{-}$ vs. $P57^{+}95^{+}$	505.9	**<0.0001**
$M57^{-}95^{+}$ vs. $M57^{+}95^{+}$	103	>0.9999
$M57^{-}95^{+}$ vs. N	35.15	>0.9999
$M57^{-}95^{+}$ vs. $N57^{-}95^{+}$	133.2	>0.9999
$M57^{-}95^{+}$ vs. $N57^{+}95^{+}$	104.8	>0.9999
$M57^{-}95^{+}$ vs. O	64.43	>0.9999
$M57^{-}95^{+}$ vs. $O57^{-}95^{+}$	20.6	>0.9999
$M57^{-}95^{+}$ vs. $O57^{+}95^{+}$	94.36	>0.9999
$M57^{-}95^{+}$ vs. P	156.3	>0.9999
$M57^{-}95^{+}$ vs. $P57^{-}95^{+}$	184.2	>0.9999
$M57^{-}95^{+}$ vs. $P57^{+}95^{+}$	239.9	>0.9999
$M57^{+}95^{+}$ vs. N	−67.85	>0.9999
$M57^{+}95^{+}$ vs. $N57^{-}95^{+}$	30.23	>0.9999
$M57^{+}95^{+}$ vs. $N57^{+}95^{+}$	1.822	>0.9999
$M57^{+}95^{+}$ vs. O	−38.58	>0.9999
$M57^{+}95^{+}$ vs. $O57^{-}95^{+}$	−82.4	>0.9999
$M57^{+}95^{+}$ vs. $O57^{+}95^{+}$	−8.645	>0.9999
$M57^{+}95^{+}$ vs. P	53.29	>0.9999
$M57^{+}95^{+}$ vs. $P57^{-}95^{+}$	81.21	>0.9999
$M57^{+}95^{+}$ vs. $P57^{+}95^{+}$	136.9	>0.9999
N vs. $N57^{-}95^{+}$	98.09	>0.9999
N vs. $N57^{+}95^{+}$	69.67	>0.9999
N vs. O	29.28	>0.9999
N vs. $O57^{-}95^{+}$	−14.55	>0.9999

N vs. O57^{+}95^{+}	59.21	>0.9999
N vs. P	121.1	>0.9999
N vs. P57^{-}95^{+}	149.1	>0.9999
N vs. P57^{+}95^{+}	204.7	0.7764
N57^{-}95^{+} vs. N57^{+}95^{+}	−28.41	>0.9999
N57^{-}95^{+} vs. O	−68.81	>0.9999
N57^{-}95^{+} vs. O57^{-}95^{+}	−112.6	>0.9999
N57^{-}95^{+} vs. O57^{+}95^{+}	−38.88	>0.9999
N57^{-}95^{+} vs. P	23.05	>0.9999
N57^{-}95^{+} vs. P57^{-}95^{+}	50.97	>0.9999
N57^{-}95^{+} vs. P57^{+}95^{+}	106.6	>0.9999
N57^{+}95^{+} vs. O	−40.4	>0.9999
N57^{+}95^{+} vs. O57^{-}95^{+}	−84.22	>0.9999
N57^{+}95^{+} vs. O57^{+}95^{+}	−10.47	>0.9999
N57^{+}95^{+} vs. P	51.46	>0.9999
N57^{+}95^{+} vs. P57^{-}95^{+}	79.39	>0.9999
N57^{+}95^{+} vs. P57^{+}95^{+}	135.1	>0.9999
O vs. O57^{-}95^{+}	−43.82	>0.9999
O vs. O57^{+}95^{+}	29.93	>0.9999
O vs. P	91.86	>0.9999
O vs. P57^{-}95^{+}	119.8	>0.9999
O vs. P57^{+}95^{+}	175.5	>0.9999
O57^{-}95^{+} vs. O57^{+}95^{+}	73.75	>0.9999
O57^{-}95^{+} vs. P	135.7	>0.9999
O57^{-}95^{+} vs. P57^{-}95^{+}	163.6	>0.9999
O57^{-}95^{+} vs. P57^{+}95^{+}	219.3	>0.9999
O57^{+}95^{+} vs. P	61.93	>0.9999
O57^{+}95^{+} vs. P57^{-}95^{+}	89.85	>0.9999
O57^{+}95^{+} vs. P57^{+}95^{+}	145.5	>0.9999
P vs. P57^{-}95^{+}	27.92	>0.9999
P vs. P57^{+}95^{+}	83.6	>0.9999
P57^{-}95^{+} vs. P57^{+}95^{+}	55.67	>0.9999
**Donor 5**		
Naive vs. J	435	**<0.0001**
Naive vs. J57^{-}95^{+}	436.1	**<0.0001**
Naive vs. J57^{+}95^{+}	382.9	**<0.0001**
Naive vs. K	467.9	**<0.0001**
Naive vs. K57^{-}95^{-}	157.2	>0.9999
Naive vs. K57^{-}95^{+}	454.8	**<0.0001**
Naive vs. K57^{+}95^{+}	261.7	>0.9999
Naive vs. L	−22.36	>0.9999
Naive vs. L57^{-}95^{-}	−18.38	>0.9999
Naive vs. L57^{-}95^{+}	118.9	>0.9999
Naive vs. M	127.8	>0.9999
Naive vs. M57^{-}95^{-}	83.15	>0.9999
Naive vs. M57^{-}95^{+}	267.3	0.0622
Naive vs. M57^{+}95^{+}	294.9	0.2643
Naive vs. N	392.9	**<0.0001**
Naive vs. N57^{-}95^{+}	461.4	**<0.0001**
Naive vs. N57^{+}95^{+}	354.7	**0.0002**
Naive vs. O	372	**<0.0001**
Naive vs. O57^{-}95^{+}	470.5	**<0.0001**
Naive vs. O57^{+}95^{+}	291.3	**0.0243**

Naive vs. P	497.2	**<0.0001**
Naive vs. P57^{-}95^{+}	470.8	**<0.0001**
Naive vs. P57^{+}95^{+}	514.1	**<0.0001**
J vs. J57^{-}95^{+}	1.053	>0.9999
J vs. J57^{+}95^{+}	−52.1	>0.9999
J vs. K	32.87	>0.9999
J vs. K57^{-}95^{-}	−277.8	**0.0114**
J vs. K57^{-}95^{+}	19.74	>0.9999
J vs. K57^{+}95^{+}	−173.3	>0.9999
J vs. L	−457.4	**<0.0001**
J vs. L57^{-}95^{-}	−453.4	**<0.0001**
J vs. L57^{-}95^{+}	−316.2	**0.0075**
J vs. M	−307.2	**0.0284**
J vs. M57^{-}95^{-}	−351.9	**0.0004**
J vs. M57^{-}95^{+}	−167.8	>0.9999
J vs. M57^{+}95^{+}	−140.1	>0.9999
J vs. N	−42.09	>0.9999
J vs. N57^{-}95^{+}	26.41	>0.9999
J vs. N57^{+}95^{+}	−80.38	>0.9999
J vs. O	−63	>0.9999
J vs. O57^{-}95^{+}	35.47	>0.9999
J vs. O57^{+}95^{+}	−143.7	>0.9999
J vs. P	62.18	>0.9999
J vs. P57^{-}95^{+}	35.74	>0.9999
J vs. P57^{+}95^{+}	79.03	>0.9999
J57^{-}95^{+} vs. J57^{+}95^{+}	−53.15	>0.9999
J57^{-}95^{+} vs. K	31.81	>0.9999
J57^{-}95^{+} vs. K57^{-}95^{-}	−278.8	**0.0131**
J57^{-}95^{+} vs. K57^{-}95^{+}	18.69	>0.9999
J57^{-}95^{+} vs. K57^{+}95^{+}	−174.4	>0.9999
J57^{-}95^{+} vs. L	−458.4	**<0.0001**
J57^{-}95^{+} vs. L57^{-}95^{-}	−454.5	**<0.0001**
J57^{-}95^{+} vs. L57^{-}95^{+}	−317.2	**0.0084**
J57^{-}95^{+} vs. M	−308.3	**0.0307**
J57^{-}95^{+} vs. M57^{-}95^{-}	−352.9	**0.0005**
J57^{-}95^{+} vs. M57^{-}95^{+}	−168.8	>0.9999
J57^{-}95^{+} vs. M57^{+}95^{+}	−141.2	>0.9999
J57^{-}95^{+} vs. N	−43.14	>0.9999
J57^{-}95^{+} vs. N57^{-}95^{+}	25.36	>0.9999
J57^{-}95^{+} vs. N57^{+}95^{+}	−81.43	>0.9999
J57^{-}95^{+} vs. O	−64.05	>0.9999
J57^{-}95^{+} vs. O57^{-}95^{+}	34.42	>0.9999
J57^{-}95^{+} vs. O57^{+}95^{+}	−144.7	>0.9999
J57^{-}95^{+} vs. P	61.13	>0.9999
J57^{-}95^{+} vs. P57^{-}95^{+}	34.69	>0.9999
J57^{-}95^{+} vs. P57^{+}95^{+}	77.97	>0.9999
J57^{+}95^{+} vs. K	84.97	>0.9999
J57^{+}95^{+} vs. K57^{-}95^{-}	−225.7	0.0657
J57^{+}95^{+} vs. K57^{-}95^{+}	71.84	>0.9999
J57^{+}95^{+} vs. K57^{+}95^{+}	−121.2	>0.9999
J57^{+}95^{+} vs. L	−405.3	**<0.0001**
J57^{+}95^{+} vs. L57^{-}95^{-}	−401.3	**<0.0001**
J57^{+}95^{+} vs. L57^{-}95^{+}	−264.1	**0.0419**

$J57^{+}95^{+}$ vs. M	−255.1	0.1492
$J57^{+}95^{+}$ vs. $M57^{-}95^{-}$	−299.8	**0.0024**
$J57^{+}95^{+}$ vs. $M57^{-}95^{+}$	−115.7	>0.9999
$J57^{+}95^{+}$ vs. $M57^{+}95^{+}$	−88.04	>0.9999
$J57^{+}95^{+}$ vs. N	10.01	>0.9999
$J57^{+}95^{+}$ vs. $N57^{-}95^{+}$	78.51	>0.9999
$J57^{+}95^{+}$ vs. $N57^{+}95^{+}$	−28.28	>0.9999
$J57^{+}95^{+}$ vs. O	−10.9	>0.9999
$J57^{+}95^{+}$ vs. $O57^{-}95^{+}$	87.57	>0.9999
$J57^{+}95^{+}$ vs. $O57^{+}95^{+}$	−91.59	>0.9999
$J57^{+}95^{+}$ vs. P	114.3	>0.9999
$J57^{+}95^{+}$ vs. $P57^{-}95^{+}$	87.84	>0.9999
$J57^{+}95^{+}$ vs. $P57^{+}95^{+}$	131.1	>0.9999
K vs. $K57^{-}95^{-}$	−310.6	**0.0076**
K vs. $K57^{-}95^{+}$	−13.13	>0.9999
K vs. $K57^{+}95^{+}$	−206.2	>0.9999
K vs. L	−490.3	**<0.0001**
K vs. $L57^{-}95^{-}$	−486.3	**<0.0001**
K vs. $L57^{-}95^{+}$	−349	**0.0046**
K vs. M	−340.1	**0.0160**
K vs. $M57^{-}95^{-}$	−384.7	**0.0003**
K vs. $M57^{-}95^{+}$	−200.6	>0.9999
K vs. $M57^{+}95^{+}$	−173	>0.9999
K vs. N	−74.95	>0.9999
K vs. $N57^{-}95^{+}$	−6.455	>0.9999
K vs. $N57^{+}95^{+}$	−113.2	>0.9999
K vs. O	−95.87	>0.9999
K vs. $O57^{-}95^{+}$	2.608	>0.9999
K vs. $O57^{+}95^{+}$	−176.6	>0.9999
K vs. P	29.32	>0.9999
K vs. $P57^{-}95^{+}$	2.876	>0.9999
K vs. $P57^{+}95^{+}$	46.16	>0.9999
$K57^{-}95^{-}$ vs. $K57^{-}95^{+}$	297.5	**0.0177**
$K57^{-}95^{-}$ vs. $K57^{+}95^{+}$	104.4	>0.9999
$K57^{-}95^{-}$ vs. L	−179.6	>0.9999
$K57^{-}95^{-}$ vs. $L57^{-}95^{-}$	−175.6	>0.9999
$K57^{-}95^{-}$ vs. $L57^{-}95^{+}$	−38.39	>0.9999
$K57^{-}95^{-}$ vs. M	−29.46	>0.9999
$K57^{-}95^{-}$ vs. $M57^{-}95^{-}$	−74.09	>0.9999
$K57^{-}95^{-}$ vs. $M57^{-}95^{+}$	110	>0.9999
$K57^{-}95^{-}$ vs. $M57^{+}95^{+}$	137.6	>0.9999
$K57^{-}95^{-}$ vs. N	235.7	0.8499
$K57^{-}95^{-}$ vs. $N57^{-}95^{+}$	304.2	**0.0030**
$K57^{-}95^{-}$ vs. $N57^{+}95^{+}$	197.4	>0.9999
$K57^{-}95^{-}$ vs. O	214.8	>0.9999
$K57^{-}95^{-}$ vs. $O57^{-}95^{+}$	313.3	**0.0046**
$K57^{-}95^{-}$ vs. $O57^{+}95^{+}$	134.1	>0.9999
$K57^{-}95^{-}$ vs. P	340	**0.0006**
$K57^{-}95^{-}$ vs. $P57^{-}95^{+}$	313.5	**0.0036**
$K57^{-}95^{-}$ vs. $P57^{+}95^{+}$	356.8	**0.0008**
$K57^{-}95^{+}$ vs. $K57^{+}95^{+}$	−193.1	>0.9999
$K57^{-}95^{+}$ vs. L	−477.1	**<0.0001**
$K57^{-}95^{+}$ vs. $L57^{-}95^{-}$	−473.1	**<0.0001**

K57^{-}95^{+} vs. L57^{-}95^{+}	−335.9	**0.0102**
K57^{-}95^{+} vs. M	−327	**0.0324**
K57^{-}95^{+} vs. M57^{-}95^{-}	−371.6	**0.0008**
K57^{-}95^{+} vs. M57^{-}95^{+}	−187.5	>0.9999
K57^{-}95^{+} vs. M57^{+}95^{+}	−159.9	>0.9999
K57^{-}95^{+} vs. N	−61.83	>0.9999
K57^{-}95^{+} vs. N57^{-}95^{+}	6.671	>0.9999
K57^{-}95^{+} vs. N57^{+}95^{+}	−100.1	>0.9999
K57^{-}95^{+} vs. O	−82.74	>0.9999
K57^{-}95^{+} vs. O57^{-}95^{+}	15.73	>0.9999
K57^{-}95^{+} vs. O57^{+}95^{+}	−163.4	>0.9999
K57^{-}95^{+} vs. P	42.44	>0.9999
K57^{-}95^{+} vs. P57^{-}95^{+}	16	>0.9999
K57^{-}95^{+} vs. P57^{+}95^{+}	59.29	>0.9999
K57^{+}95^{+} vs. L	−284	>0.9999
K57^{+}95^{+} vs. L57^{-}95^{-}	−280.1	>0.9999
K57^{+}95^{+} vs. L57^{-}95^{+}	−142.8	>0.9999
K57^{+}95^{+} vs. M	−133.9	>0.9999
K57^{+}95^{+} vs. M57^{-}95^{-}	−178.5	>0.9999
K57^{+}95^{+} vs. M57^{-}95^{+}	5.583	>0.9999
K57^{+}95^{+} vs. M57^{+}95^{+}	33.21	>0.9999
K57^{+}95^{+} vs. N	131.3	>0.9999
K57^{+}95^{+} vs. N57^{-}95^{+}	199.8	>0.9999
K57^{+}95^{+} vs. N57^{+}95^{+}	92.97	>0.9999
K57^{+}95^{+} vs. O	110.3	>0.9999
K57^{+}95^{+} vs. O57^{-}95^{+}	208.8	>0.9999
K57^{+}95^{+} vs. O57^{+}95^{+}	29.66	>0.9999
K57^{+}95^{+} vs. P	235.5	>0.9999
K57^{+}95^{+} vs. P57^{-}95^{+}	209.1	>0.9999
K57^{+}95^{+} vs. P57^{+}95^{+}	252.4	>0.9999
L vs. L57^{-}95^{-}	3.977	>0.9999
L vs. L57^{-}95^{+}	141.2	>0.9999
L vs. M	150.1	>0.9999
L vs. M57^{-}95^{-}	105.5	>0.9999
L vs. M57^{-}95^{+}	289.6	0.5618
L vs. M57^{+}95^{+}	317.3	0.8668
L vs. N	415.3	**0.0027**
L vs. N57^{-}95^{+}	483.8	**<0.0001**
L vs. N57^{+}95^{+}	377	**0.0140**
L vs. O	394.4	**0.0048**
L vs. O57^{-}95^{+}	492.9	**<0.0001**
L vs. O57^{+}95^{+}	313.7	0.2752
L vs. P	519.6	**<0.0001**
L vs. P57^{-}95^{+}	493.1	**<0.0001**
L vs. P57^{+}95^{+}	536.4	**<0.0001**
L57^{-}95^{-} vs. L57^{-}95^{+}	137.2	>0.9999
L57^{-}95^{-} vs. M	146.2	>0.9999
L57^{-}95^{-} vs. M57^{-}95^{-}	101.5	>0.9999
L57^{-}95^{-} vs. M57^{-}95^{+}	285.6	0.3027
L57^{-}95^{-} vs. M57^{+}95^{+}	313.3	0.5812
L57^{-}95^{-} vs. N	411.3	**0.0007**
L57^{-}95^{-} vs. N57^{-}95^{+}	479.8	>0.9999
L57^{-}95^{-} vs. N57^{+}95^{+}	373	>0.9999

$L57^{-}95^{-}$ vs. O	390.4	>0.9999
$L57^{-}95^{-}$ vs. $O57^{-}95^{+}$	488.9	>0.9999
$L57^{-}95^{-}$ vs. $O57^{+}95^{+}$	309.7	>0.9999
$L57^{-}95^{-}$ vs. P	515.6	>0.9999
$L57^{-}95^{-}$ vs. $P57^{-}95^{+}$	489.2	>0.9999
$L57^{-}95^{-}$ vs. $P57^{+}95^{+}$	532.4	>0.9999
$L57^{-}95^{+}$ vs. M	8.928	>0.9999
$L57^{-}95^{+}$ vs. $M57^{-}95^{-}$	−35.7	>0.9999
$L57^{-}95^{+}$ vs. $M57^{-}95^{+}$	148.4	>0.9999
$L57^{-}95^{+}$ vs. $M57^{+}95^{+}$	176	>0.9999
$L57^{-}95^{+}$ vs. N	274.1	>0.9999
$L57^{-}95^{+}$ vs. $N57^{-}95^{+}$	342.6	>0.9999
$L57^{-}95^{+}$ vs. $N57^{+}95^{+}$	235.8	>0.9999
$L57^{-}95^{+}$ vs. O	253.2	>0.9999
$L57^{-}95^{+}$ vs. $O57^{-}95^{+}$	351.6	>0.9999
$L57^{-}95^{+}$ vs. $O57^{+}95^{+}$	172.5	>0.9999
$L57^{-}95^{+}$ vs. P	378.4	>0.9999
$L57^{-}95^{+}$ vs. $P57^{-}95^{+}$	351.9	>0.9999
$L57^{-}95^{+}$ vs. $P57^{+}95^{+}$	395.2	>0.9999
M vs. $M57^{-}95^{-}$	−44.63	>0.9999
M vs. $M57^{-}95^{+}$	139.5	>0.9999
M vs. $M57^{+}95^{+}$	167.1	>0.9999
M vs. N	265.2	>0.9999
M vs. $N57^{-}95^{+}$	333.7	>0.9999
M vs. $N57^{+}95^{+}$	226.9	>0.9999
M vs. O	244.2	>0.9999
M vs. $O57^{-}95^{+}$	342.7	>0.9999
M vs. $O57^{+}95^{+}$	163.6	>0.9999
M vs. P	369.4	>0.9999
M vs. $P57^{-}95^{+}$	343	>0.9999
M vs. $P57^{+}95^{+}$	386.3	**<0.0001**
$M57^{-}95^{-}$ vs. $M57^{-}95^{+}$	184.1	**<0.0001**
$M57^{-}95^{-}$ vs. $M57^{+}95^{+}$	211.7	**<0.0001**
$M57^{-}95^{-}$ vs. N	309.8	**<0.0001**
$M57^{-}95^{-}$ vs. $N57^{-}95^{+}$	378.3	>0.9999
$M57^{-}95^{-}$ vs. $N57^{+}95^{+}$	271.5	**<0.0001**
$M57^{-}95^{-}$ vs. O	288.9	>0.9999
$M57^{-}95^{-}$ vs. $O57^{-}95^{+}$	387.4	>0.9999
$M57^{-}95^{-}$ vs. $O57^{+}95^{+}$	208.2	>0.9999
$M57^{-}95^{-}$ vs. P	414.1	>0.9999
$M57^{-}95^{-}$ vs. $P57^{-}95^{+}$	387.6	>0.9999
$M57^{-}95^{-}$ vs. $P57^{+}95^{+}$	430.9	>0.9999
$M57^{-}95^{+}$ vs. $M57^{+}95^{+}$	27.62	0.0622
$M57^{-}95^{+}$ vs. N	125.7	0.2643
$M57^{-}95^{+}$ vs. $N57^{-}95^{+}$	194.2	**<0.0001**
$M57^{-}95^{+}$ vs. $N57^{+}95^{+}$	87.38	**<0.0001**
$M57^{-}95^{+}$ vs. O	104.8	**0.0002**
$M57^{-}95^{+}$ vs. $O57^{-}95^{+}$	203.2	**<0.0001**
$M57^{-}95^{+}$ vs. $O57^{+}95^{+}$	24.07	**<0.0001**
$M57^{-}95^{+}$ vs. P	229.9	**0.0243**
$M57^{-}95^{+}$ vs. $P57^{-}95^{+}$	203.5	**<0.0001**
$M57^{-}95^{+}$ vs. $P57^{+}95^{+}$	246.8	**<0.0001**
$M57^{+}95^{+}$ vs. N	98.05	**<0.0001**

M57^{+}95^{+} vs. N57^{-}95^{+}	166.5	>0.9999
M57^{+}95^{+} vs. N57^{+}95^{+}	59.76	>0.9999
M57^{+}95^{+} vs. O	77.14	>0.9999
M57^{+}95^{+} vs. O57^{-}95^{+}	175.6	**0.0114**
M57^{+}95^{+} vs. O57^{+}95^{+}	−3.55	>0.9999
M57^{+}95^{+} vs. P	202.3	>0.9999
M57^{+}95^{+} vs. P57^{-}95^{+}	175.9	**<0.0001**
M57^{+}95^{+} vs. P57^{+}95^{+}	219.2	**<0.0001**
N vs. N57^{-}95^{+}	68.5	**0.0075**
N vs. N57^{+}95^{+}	−38.29	**0.0284**
N vs. O	−20.91	**0.0004**
N vs. O57^{-}95^{+}	77.56	>0.9999
N vs. O57^{+}95^{+}	−101.6	>0.9999
N vs. P	104.3	>0.9999
N vs. P57^{-}95^{+}	77.83	>0.9999
N vs. P57^{+}95^{+}	121.1	>0.9999
N57^{-}95^{+} vs. N57^{+}95^{+}	−106.8	>0.9999
N57^{-}95^{+} vs. O	−89.41	>0.9999
N57^{-}95^{+} vs. O57^{-}95^{+}	9.063	>0.9999
N57^{-}95^{+} vs. O57^{+}95^{+}	−170.1	>0.9999
N57^{-}95^{+} vs. P	35.77	>0.9999
N57^{-}95^{+} vs. P57^{-}95^{+}	9.331	>0.9999
N57^{-}95^{+} vs. P57^{+}95^{+}	52.62	>0.9999
N57^{+}95^{+} vs. O	17.38	>0.9999
N57^{+}95^{+} vs. O57^{-}95^{+}	115.9	**0.0131**
N57^{+}95^{+} vs. O57^{+}95^{+}	−63.31	>0.9999
N57^{+}95^{+} vs. P	142.6	>0.9999
N57^{+}95^{+} vs. P57^{-}95^{+}	116.1	**<0.0001**
N57^{+}95^{+} vs. P57^{+}95^{+}	159.4	**<0.0001**
O vs. O57^{-}95^{+}	98.47	**0.0084**
O vs. O57^{+}95^{+}	−80.69	**0.0307**
O vs. P	125.2	**0.0005**
O vs. P57^{-}95^{+}	98.74	>0.9999
O vs. P57^{+}95^{+}	142	>0.9999
O57^{-}95^{+} vs. O57^{+}95^{+}	−179.2	>0.9999
O57^{-}95^{+} vs. P	26.71	>0.9999
O57^{-}95^{+} vs. P57^{-}95^{+}	0.2677	>0.9999
O57^{-}95^{+} vs. P57^{+}95^{+}	43.55	>0.9999
O57^{+}95^{+} vs. P	205.9	>0.9999
O57^{+}95^{+} vs. P57^{-}95^{+}	179.4	>0.9999
O57^{+}95^{+} vs. P57^{+}95^{+}	222.7	>0.9999
P vs. P57^{-}95^{+}	−26.44	>0.9999
P vs. P57^{+}95^{+}	16.85	>0.9999
P57^{-}95^{+} vs. P57^{+}95^{+}	43.28	>0.9999

Alpha = 0.05.

Bold font indicates significance.
